# Molecular logics in dual sensor regulation of diguanylate cyclase activity—Phosphorylation OR blue‐light activation

**DOI:** 10.1002/pro.70765

**Published:** 2026-08-24

**Authors:** Maximilian Fuchs, Andreas Winkler

**Affiliations:** ^1^ Institute of Biochemistry Graz University of Technology Graz Austria; ^2^ BioTechMed‐Graz Graz Austria

**Keywords:** blue light, diguanylate cyclase, GGDEF, LOV, molecular logics, phosphorylation, Rec

## Abstract

Bacterial cells use multiple environmental cues to regulate levels of the second messenger cyclic dimeric GMP. This compound influences key lifestyle decisions such as motility, biofilm formation, or virulence. Although many diguanylate cyclases (DGCs) combined with various sensory domains have been studied previously, how distinct inputs are integrated within individual enzymes remains incompletely understood. Here, we investigate a cyanobacterial family of dual sensor DGCs that combine an N‐terminal receiver (Rec) domain followed by a light‐oxygen‐voltage (LOV) domain upstream of a diguanylate cyclase (GGDEF) domain. Using in vivo activity screening and in vitro characterization, we determined how phosphorylation and blue light, individually and jointly, regulate enzymatic activity. By measuring kinetic parameters across four defined functional states, unphosphorylated or phosphorylated, in combination with dark or light states, we reveal logic gate‐like behaviors. One representative, *La*RldC, integrates both signals with pronounced fold‐changes in activity, consistent with overall OR‐type logic and with light acting as the dominant input. Our results demonstrate its function as a molecular gate coupling phosphorylation and illumination sensing to cyclic‐di‐GMP formation. These findings provide valuable insights into multi‐signal decision‐making in cyanobacteria and establish further understanding of how modular sensory domains are wired to control bacterial second‐messenger signaling.

## 1 INTRODUCTION

Across all kingdoms of life, cells respond to changing environments by processing diverse physical and chemical cues into behavioral decisions. Among others, phosphorylation, redox state, pH or light conditions can be sensed by a range of modular protein domains (Papon & Stock, 2019; Isom et al., 2013; Antelmann & Helmann, 2011; Crosson & Moffat, 2002; Gomelsky & Klug, 2002). The interactions and cross‐talk of sensory modules with so called effector or output domains eventually enables intricate molecular mechanisms for multi‐signal integration and precisely adjusted physiological responses (Papin et al., 2005; Bhattacharyya et al., 2006; Francis & Porter, 2019).

In bacteria, the nucleotide second messenger 3′‐5′‐cyclic‐dimeric‐GMP (c‐di‐GMP) orchestrates transitions between motile and sessile lifestyles, biofilm formation, virulence, and cell cycle control (Obeng et al., 2023; Enomoto et al., 2023; Guest et al., 2022; Hengge et al., 2016; Liu et al., 2025; Nie et al., 2024; Tamayo et al., 2007). Because cellular outcomes depend on c‐di‐GMP concentration, the enzymes that synthesize and degrade this nucleotide are at the heart of cellular decision‐making. The formation of c‐di‐GMP from two molecules of guanosine‐5′‐triphosphate (GTP) is catalyzed by diguanylate cyclases (DGCs) belonging to the family of GGDEF proteins (Paul et al., 2004; Sondermann et al., 2012). Due to their central role for bacterial lifestyle decisions, a range of sensory domains have been fused to these DGCs during evolution, making them versatile natural integrators of environmental inputs. Many GGDEF enzymes carry multiple sensory modules, implying modular logic for decoding diverse stimuli. Exactly how sensory domains effectively couple to the GGDEF catalytic core and tune enzymatic parameters or the oligomeric state, however, remains incompletely understood, particularly in multi‐sensor architectures.

Responding to a range of different signal inputs is especially important for cyanobacteria as this lineage experiences pronounced diel transitions due to photosynthesis–respiration cycles that result in oscillations in intracellular pH and redox state (Guo et al., 2014; Mangan et al., 2016; Welkie et al., 2019). These variables, together with changing light conditions, compose a characteristic input landscape that is expected to also impinge on the activity of the frequently occurring DGCs in cyanobacteria. Indeed, cyanobacteria feature a range of light‐regulated GGDEF architectures (Agostoni et al., 2013; Enomoto et al., 2015; Enomoto et al., 2023; Savakis et al., 2012). In this study, we characterize a small subgroup of dual sensor DGCs combining an N‐terminal Receiver (Rec) domain and a central sensor of light‐oxygen‐voltage (LOV) domain; a combination that, to our current knowledge, is confined to cyanobacteria. Such Rec‐LOV‐DGC systems (termed RldCs from here on) might serve as interesting examples of how molecular logics can be realized on a single polypeptide chain.

Rec and LOV domains are frequently occurring sensory systems that respond to phosphorylation and blue light, respectively. Rec domains typically are part of two component systems (Gao et al., 2019), where they get phosphorylated on a conserved aspartate residue by cognate histidine kinases. In turn, they fulfill their action as response regulator and initiate cellular responses by, for example, tuning transcription factor binding to target gene promoter sequences (Xie et al., 2022). In the case of RldCs, the N‐terminal positioning of the Rec domain relative to another sensory element, the LOV domain, raises interesting questions about how Rec activation would influence blue light processing in the photosensory part. Typically, the flavin cofactor bound to LOV domains is activated by blue light and forms a characteristic photoadduct with a nearby cysteine residue in the cofactor binding pocket (Crosson & Moffat, 2002; Swartz et al., 2001). Ultimately, these local rearrangements trigger conformational changes that can be transmitted to a variety of downstream effector domains (Glantz et al., 2016), frequently via helical connectors (Swingle et al., 2025; Vide et al., 2023; Zayner et al., 2012). In cyanobacteria, light strongly impacts pH and the redox state of the cell; thereby illumination might even result in extended multimodal regulation regimes via indirect effects including protonation of residues and/or thiol chemistry.

**FIGURE 1:**
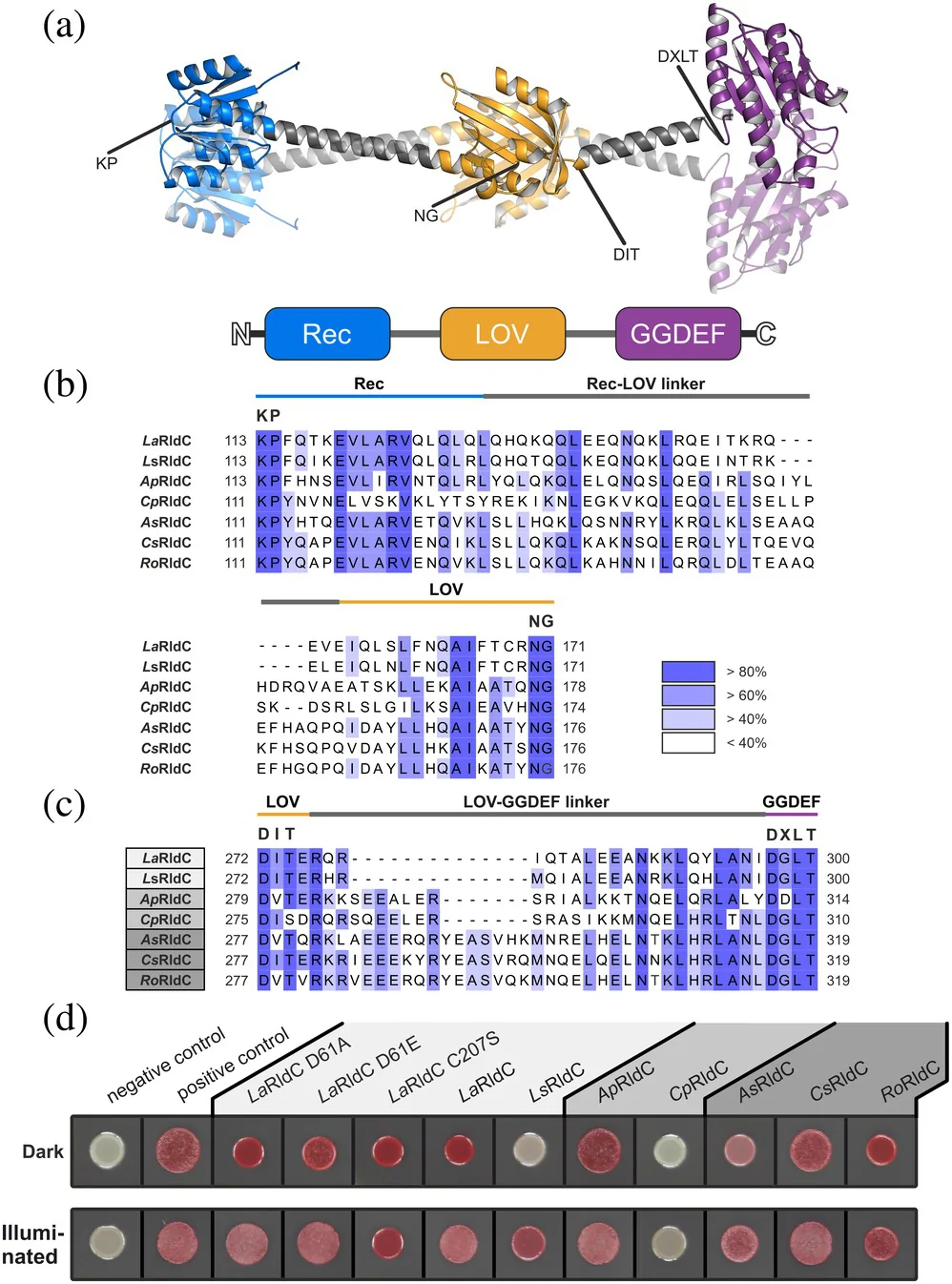
Domain architecture, linker sequence alignments and in vivo activity assay of RldC homologs. (a) AlphaFold 2 model of *La*RldC, colored according to individual domains: Rec—blue, LOV—orange, GGDEF—violet. Linker elements between Rec and LOV as well as LOV and GGDEF are colored in gray. The individual sequence tags shown in panel (b) are highlighted. (b) Multiple sequence alignment of linker sequences between the Rec and LOV domain of selected homologs. The full sequence alignment is shown in Figure S1. The KP motif in the Rec domain marks the start of the last loop before Rec‐*α*5. NG marks the conserved start of the first beta strand (*Aβ*) following the *A*′*α* helix of the LOV domain. The legend shows color coding based on percentage identity. (c) Multiple sequence alignment highlighting the three groups with different linker lengths between LOV and GGDEF domain, by different shades of gray. The LOV‐DIT and GGDEF‐DXLT motifs flank the linker region. (d) The in vivo activity of RldC homologs was investigated using a cellular c‐di‐GMP level assay. The rdar (red, dry, rough) morphotype is characteristic for elevated c‐di‐GMP levels. The previously studied LOV‐GGDEF protein *Ms*LadC^GGAAF^ and a truncated GCN4cc‐*Ms*GGDEF act as negative and positive controls (Vide et al., 2026), respectively. Uncut image shown in Figure S14. The screening was performed in three technical replicates; one representative plate is shown.

Interestingly, RldCs feature linker sequences between their sensory modules and the GGDEF domain that suggest parallel coiled‐coils as connecting elements (Figure 1a). Such dimeric helical structures are frequently observed in systems allowing allosteric signal integration (signaling helices, coiled‐coils). Especially in the context of GGDEF domains, characteristic register switching mechanisms have been shown to play a central role in signal processing and transduction (Vide et al., 2026; Gourinchas et al., 2017; Teixeira et al., 2021; Vide et al., 2023). In two of such systems, either blue light or phosphorylation (LadC or DgcR, respectively) controls GGDEF activity via exactly this mechanism. How light and phosphorylation together can control GGDEF activation, however, remains unresolved and would be interesting to compare to previously described single input systems. Understanding molecular mechanisms of the LOV domain as central hub for signal integration will also have far‐reaching consequences for other systems featuring multi‐domain architectures in combination with LOV domains: for example phototropins (Hart & Gardner, 2021) or PAS‐LOV systems (Mawphlang et al., 2026).

In order to provide a testable framework for quantitative analysis of signal integration, we expressed, purified and biochemically characterized selected representatives of the RldC protein family. In order to describe the cross‐talk between individual inputs, we quantified kinetic parameters of several homologs in all four functionally relevant states: Rec unphosphorylated versus phosphorylated in combination with LOV in its dark versus blue light state. Based on previous observations of Rec‐ or LOV‐activated GGDEF domains, the thereby testable hypotheses would likely be either OR (phosphorylation or light being sufficient to activate DGC activity) or AND gate functionality (LOV activation could act as an enabling second input to phosphorylation—or the other way around). In combination with a mutational inactivation of Rec (phosphorylatable Asp to Ala substitution) and LOV (FMN adduct forming Cys to Ala substitution), we assign input causalities for each domain and their interplay in the context of logic gate functionality. All together, we investigate dual sensor DGCs as molecular logic devices in which phosphorylation and light inputs are integrated via allosteric coupling to control c‐di‐GMP synthesis. Among the tested proteins, we identify one promising target for future more detailed studies to fill the gap of missing quantitative rules for signal integration in these dual sensor systems and to provide insights into the molecular logics of Rec‐LOV‐DGCs. Our study also touches upon rules by which phosphorylation and light might integrate with pH and redox changes, which due to the cyanobacterial origin of RldCs might be of additional relevance.

Defining integration rules in dual sensor GGDEF enzymes advances our understanding of c‐di‐GMP signaling architectures that enable nuanced environmental decision‐making. These principles can be applied to engineer light‐ and phosphorylation‐gated cyclases, offering precise control over bacterial behaviors and potentially novel optogenetic tools. Understanding how phosphorylation of the Rec domain influences light control of c‐di‐GMP synthesis could be employed to generate logic gates that can additionally integrate chemical cues allowing fine tuning of cellular effects. Conceptually, such systems may realize AND, OR, or even antagonistic (“NAND” or “NOR”) logic depending on the sign and magnitude of the coupling between Rec, LOV and the target output domain. As far as significance and broader impact are concerned, understanding dual‐input integration has implications for bacterial decision‐making processes, spatial and temporal control of the bacterial second messenger c‐di‐GMP, and future synthetic biology/optogenetics approaches.

## 2 RESULTS

Using InterPro and PSI‐BLAST, we gathered an initial set of approximately 1500 sequences featuring a Rec‐PAS‐GGDEF architecture. We filtered these by looking specifically for Rec‐LOV‐GGDEF architectures, ensuring the presence of specific conserved residues for individual domains: Rec—conserved aspartate as phosphorylation site, LOV—GX(N/D)C(R/H)(F/I)L(Q/A) motif containing the adduct‐forming cysteine, GGDEF—GG(D/E)EF motif. The largest number of sequences was removed by filtering for the LOV motif; only around 10% of the sequences contained the canonical motif and were considered to act as blue‐light receptors. A few sequences were removed due to additional PAS or EAL domains, highlighting that even more complex domain architectures can be found in nature. Eventually, the reduced alignment of RldC homologs satisfying all constraints retained 15 sequences (Figure S1 and Table S1). Using AlphaFold2 predictions, we identified helical linkers connecting individual protein domains (Figure 1a). The linker between Rec and LOV domain extends the *α*5 helix of the Rec domain and connects it to the *A*′*α* helix of the LOV domain (Bourret, 2010; Glantz et al., 2016). Figure 1b shows a sequence alignment starting at the conserved KP motif in the Rec domain and ending at two conserved residues placed at the beginning of the first beta‐strand (*Aβ*) of the LOV domain. We identified characteristic linker length differences with 7‐residue offsets, as frequently observed in coiled‐coil linkers and their heptad repeats (Moutevelis & Woolfson, 2009; Truebestein & Leonard, 2016). The linker between the LOV and GGDEF domains also shows similar variability (Figure 1c), with length differences of one or two heptad repeats. Since different linker lengths have previously been shown to impact fold‐changes of enzymatic activity in diguanylate cyclase effectors (Vide et al., 2023) we selected two homologs per linker length for a more detailed characterization.

### 2.1 In vivo screening

After cloning the genes into a pET‐M11 expression vector, we used *Escherichia coli* BL21 (DE3) to perform an initial in vivo DGC activity screening: one, to assess the overall activity, and two, to estimate the influence of blue light. The screening is based on the production of cellulose and amyloids due to increased c‐di‐GMP levels in *E. coli*. The Congo red dye incorporated into the culture media accumulates at cellulose and amyloid fibers, resulting in a distinct visual appearance due to the so‐called rdar (red, dry, rough) morphotype (Roger & Cimdins, 2017). While the assay can be used to address overall diguanylate cyclase activity as well as up‐ or downregulation caused by blue light, the influence of different expression levels or proper folding cannot be easily assessed. However, as an initial qualitative analysis, it can be a valuable approach for mid‐ to high‐throughput screening. In addition to positive and negative controls from an established blue light‐activated diguanylate cyclase (Vide et al., 2026), we included additional controls in the screening to better interpret morphological changes. The variant *La*RldC D61A removes the phosphorylatable Asp, while a D61E variant attempts to mimic the phosphorylated state, as demonstrated for some receiver domains (Klose et al., 1993; Kurepa et al., n.d.). In addition, a C207S variant removes the Cys residue forming the characteristic FMN‐adduct of the LOV photocycle, thereby abolishing the typical light response. The screening results showed that the morphotype of individual homologs varies significantly (Figure 1d). We categorized them based on the observed effects. (1) Promising (*As*RldC, *La*RldC, and *Ls*RldC): While basal activity varied substantially, we observed pronounced differences between dark and illuminated plates. (2) Neutral (*Ap*RldC, *Cs*RldC, and *Ro*RldC): All showed diguanylate cyclase activity, but no apparent blue light regulation. However, for *Ap*RldC and *Cs*RldC, high expression levels could mask a light response. (3) No signal (*Cp*RldC): The morphotype of this homolog resembled the negative control. While this could mean that the protein is not producing any c‐di‐GMP, we cannot exclude other factors linked to the previously mentioned drawbacks of the screening. Since the assignment of individual homologs to these categories did not correlate with linker length variations, we proceeded to expression and purification for a more detailed in vitro characterization.

### 2.2 Challenges in protein production and purification

We produced all homologs using the corresponding codon optimized coding sequences cloned into the pET‐M11 vector and *E. coli* BL21 (DE3). Although all genes were overexpressed in *E. coli* (Figure S2), we were unable to identify suitable conditions for extracting and purifying *As*RldC and *Cs*RldC. This was most likely due to the formation of inclusion bodies. Given the complex domain architecture and the successful purification of other homologs, we did not pursue additional refolding attempts and instead focused on characterizing the proteins we purified in soluble form. Eventually, intact and native MS measurements were used to confirm the molecular weight, oligomeric state, and cofactor binding of purified samples (Table S2).

During purification and protein quantification, we observed a shoulder at the 280 nm absorption of *Ap*RldC, *La*RldC, and *Ro*RldC. Guanosine nucleotides typically feature a spectrum with a maximum around 254 nm that matches the observed shoulder and, hence, we attributed these signatures as bound c‐di‐GMP (Figure S3A). To remove c‐di‐GMP, we successfully used the EAL‐phosphodiesterase RocR (Rao et al., 2008). Complete removal of c‐di‐GMP was confirmed via HPLC (Figure S4), and also by the characteristic spectral changes in the absorption spectra of product‐free protein stocks (Figure S3B). However, in the case of *Ap*RldC, removal of c‐di‐GMP led to protein aggregation and eventually precipitation. Similar observations were made for *La*RldC and *Ro*LadC, but increasing the buffer ionic strength mitigated the decreased solubility. Interestingly, removing c‐di‐GMP in the latter case led to a transition from tetramers to dimers according to size exclusion chromatography (Figure S5). In the case of *La*RldC, c‐di‐GMP removal also had functional consequences; intact mass measurements showed the formation of a disulfide bond between the two protomers of the dimeric assembly (Figure S6A). To prevent this oxidation process (Figure S6B), we supplemented the corresponding buffers with Tris(2‐carboxyethyl)phosphine (TCEP) (Table S3).

*Cp*RldC and *Ls*RldC did not show signs of bound c‐di‐GMP (Figure S3A). Both homologs showed low enzymatic activity in the in vivo screening, which suggests that cellular c‐di‐GMP levels are lower during protein production. Although *Ls*RldC shares high sequence similarity with *La*RldC (Table S4), its purification proved more challenging, again primarily due to protein aggregation. We also observed reversible disulfide formation in intact mass measurements, similar to *La*RldC (Figure S6C,D). Although the challenging behavior during purification indicated that *Ap*RldC and *Ls*RldC are not suitable for full characterization, we included them in the spectroscopic characterization to better understand peculiarities in the different photocycles.

### 2.3 Spectroscopic characterization

**FIGURE 2:**
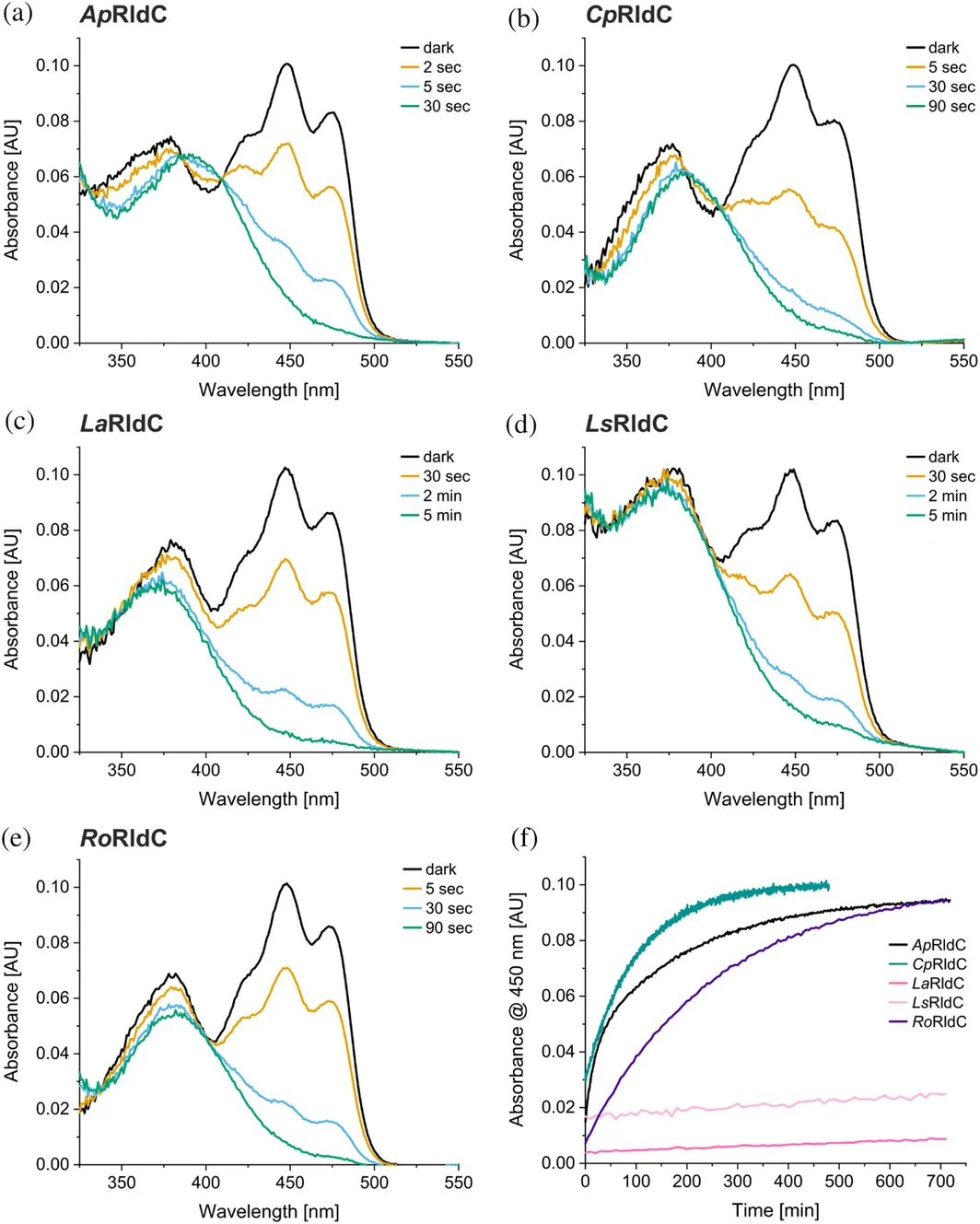
Blue light response and dark state recovery of RldC homologs. (a) *Ap*RldC shows canonical spectral changes and fast adduct formation. (b) *Cp*RldC, which also features canonical spectral changes, but slower adduct formation compared to *Ap*RldC. (c) *La*RldC with atypical light state spectrum. (d) *Ls*RldC similar to *La*RldC. Spectrum is influenced by impurities and its tendency to aggregate. (e) *Ro*RldC also shows atypical light response but with faster activation compared to *La*RldC and *Ls*RldC. (f) Dark state recovery of all characterized homologs. The 450 nm absorbance is used to follow the thermal relaxation of the photo‐adduct. Activation and thermal relaxation were measured at 20°C.

The illumination of LOV domain‐containing photoreceptors with blue light leads to characteristic spectral changes caused by the adduct formation between a conserved cysteine residue and the C4*α* of the isoalloxazine ring of the FMN cofactor. *Ap*RldC and *Cp*RldC showed LOV‐characteristic spectral changes (Figure 2a,b). Upon illumination, the 370 nm band red‐shifts to 390 nm, and the 450 nm absorption decreases. The other three homologs show what one might consider an atypical response to blue light (Figure 2c–e): the 370 nm band does not show the characteristic red‐shift. This is particularly pronounced for *La*RldC and *Ls*RldC. This is evident in the absence of the two isosbestic points, usually observed for the dark and light states, in *La*RldC, *Ls*RldC, and *Ro*RldC. During attempts to identify the molecular origin of this unexpected behavior, we observed that the wavelength of the light source significantly influences the percentage of photoproduct (Figure S7), that is, the photostationary state (PSS) (Herzog et al., 2025; Zayner & Sosnick, 2014). Two of the atypical photoproduct homologs, *La*RldC and *Ls*RldC, could only be fully activated using a 470 nm LED (Figure 2c,d), but not with the typically employed 455 nm light source. This might be caused by a more significant overlap in the near UV region of the 455 nm LED, which can induce photoconversion back to the dark state, leading to a steady state of the two populations. *Ro*RldC also benefited slightly from using the 470 nm LED.

In addition to reverse photoactivation, LOV domains also thermally revert back to their ground state. We recorded dark recovery data for all five homologs (Figure 2f) and generally observed slow thermal relaxation rates. The mean lifetimes were estimated using single‐exponential fits (Figure S8). *Cp*RldC has a mean lifetime of ~100 min and is the fastest recovering homolog. Due to its tendency to aggregate upon prolonged incubations, the corresponding trace in Figure 2f is only shown for the initial part of the measurement. Other homologs recover somewhat slower; *Ap*RldCs mean lifetime is 150 min, and *Ro*RldCs 254 min. *La*RldC and *Ls*RldC deviate substantially and exhibit particularly slow recoveries, resulting in very stable light states, preventing a fit to the dark state recovery kinetics for these two homologs. However, we demonstrated that illumination with UV light (395 nm LED) results in efficient reversion to the dark‐adapted state (Figure S9).

**FIGURE 3:**
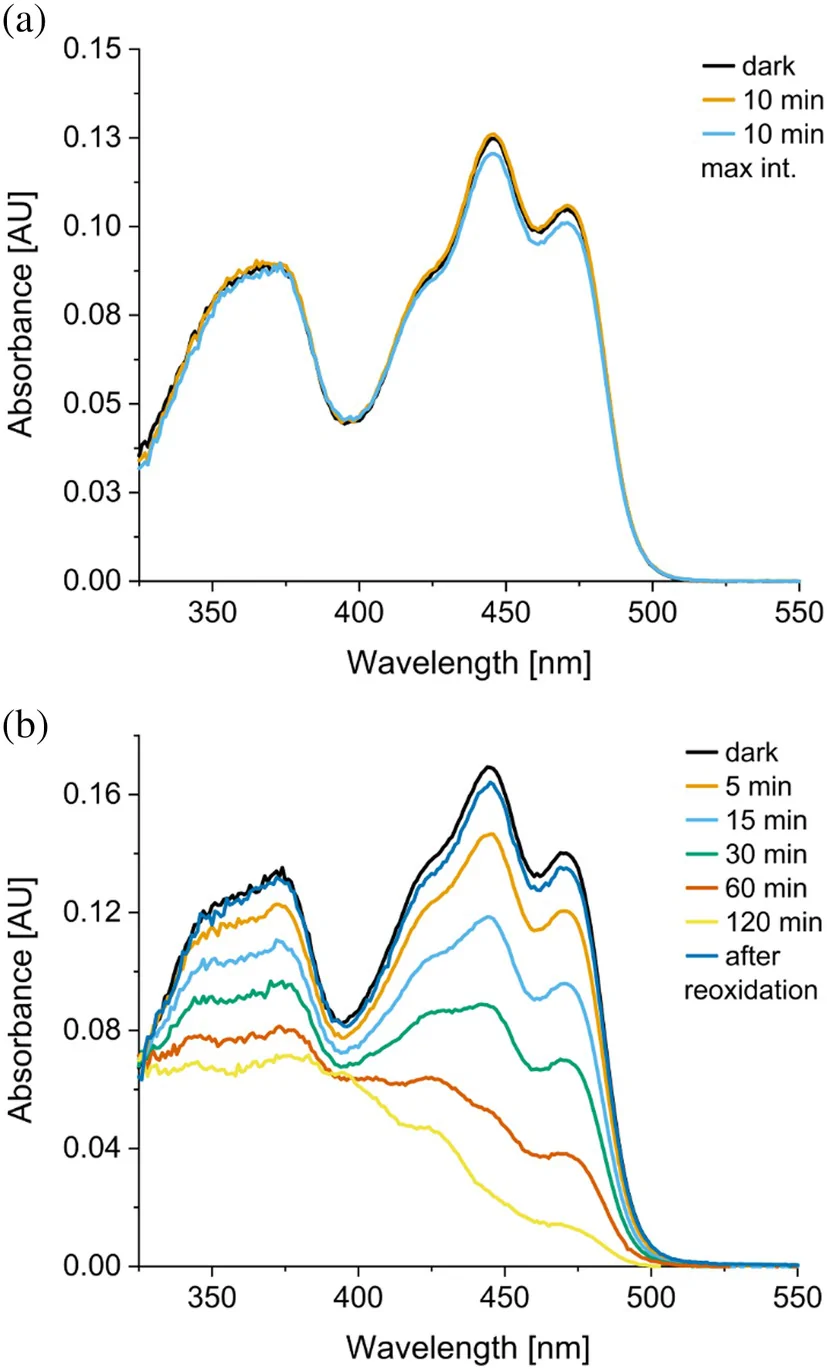
Characterization of *La*RldC C207S. (a) Absorption spectrum of *La*RldC C207S in response to illumination. (b) Photoreduction of the C207S variant using EDTA as an electron donor, combined with methyl viologen and 5‐deaza‐FAD, to increase electron transfer efficiency. FMN reoxidation, after opening the sealed quartz cuvette is also shown.

The observed atypical spectral changes raised the question of whether the affected homologs form the characteristic LOV flavin‐cysteinyl adducts. Therefore, we used the previously created C270S variant of *La*RldC, which substitutes the adduct‐forming cysteine, to check its response to illumination in vitro. The variant did not respond to blue light at comparable intensities to those used for the wild‐type protein (Figure 3a). Even illumination with maximum intensity only moderately influenced the absorption spectrum of the cofactor, actually resulting in cofactor bleaching and no recovery to the starting spectrum in the dark. In addition, we observed a broader 370 nm peak for the C207S variant compared to the wild type, but reminiscent of the supposed photoadduct signatures of *Ls*RldC and *La*RldC. To investigate if atypical photoadduct spectra could result from cofactor reduction, we performed a photoreduction under anaerobic conditions (Figure 3b). This showed that the light response of the wild type is different from the spectral changes caused by the reduction of the cofactor. The photoreduction was slow and did not proceed via semiquinone formation. Importantly, the recovery of the starting spectrum via reoxidation of the cofactor was significantly faster than the recovery of the photoadduct. Although the spectral properties of some homologs are atypical for LOV proteins, the results confirm the functionality as blue‐light photoreceptors.

### 2.4 Phosphorylation experiments

**FIGURE 4:**
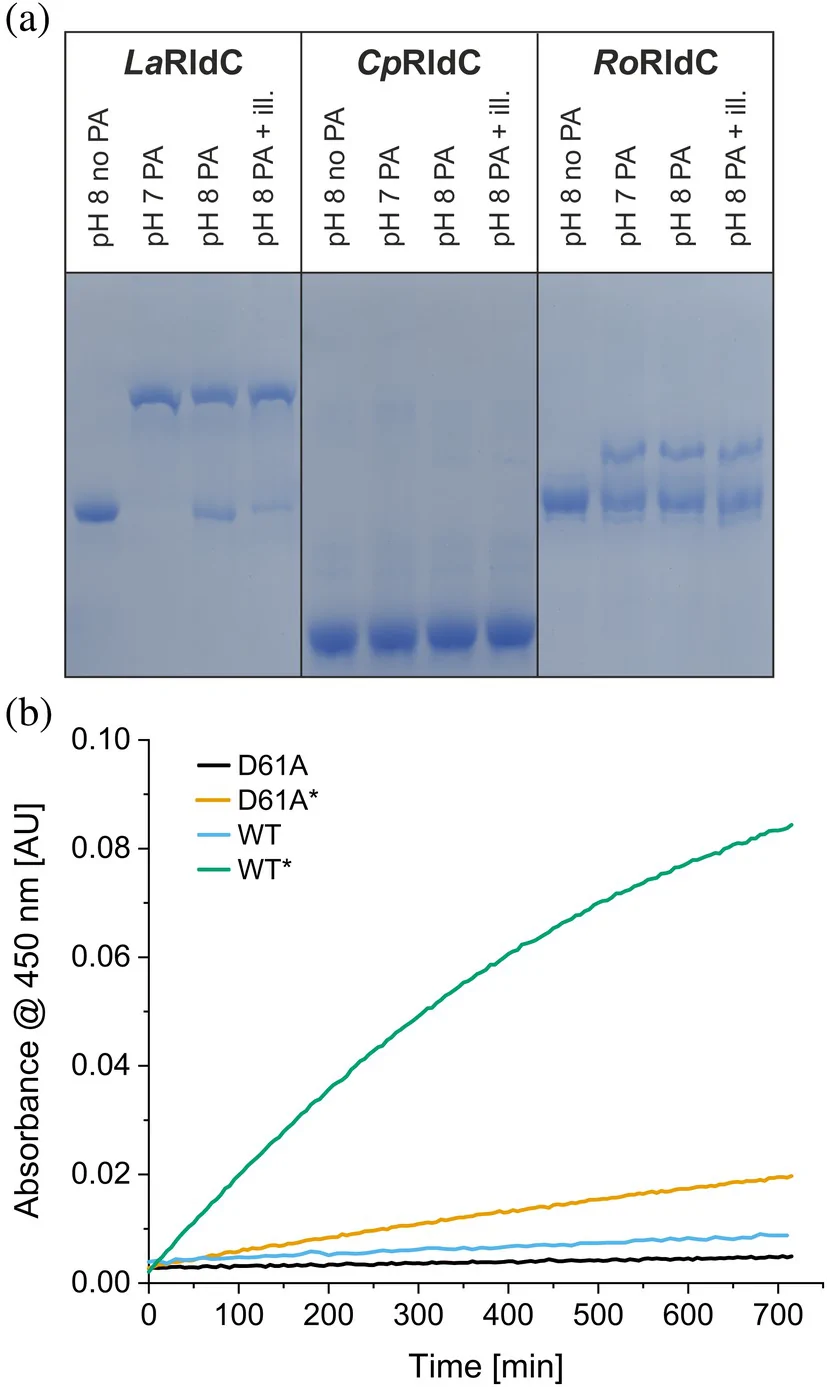
Rec domain phosphorylation and implications for LOV photocycle properties. (a) Phos‐Tag™ SDS PAGE analysis of the amount of phosphorylation after 1 h of incubation with 50 mM PA under different conditions. Unless indicated, samples were kept in the dark state during incubation. Complete SDS‐PAGE image shown in Figure S15. (b) Changes in dark state recovery of *La*RldC WT and the D61A variant before and after incubation with 50 mM PA, as indicated by an asterisk.

For the second sensory input, the Rec domain, phosphorylation is typically mediated by cognate histidine kinases, which autophosphorylate in response to diverse signals. The phosphoryl group can then be transferred to the conserved aspartate of the respective Rec domains. While this process is usually the most efficient way to phosphorylate a Rec domain (Mayover et al., 1999), small‐molecule phosphodonors such as acetylphosphate and phosphoramidate (PA) can also be employed. We used Phos‐Tag™ SDS‐PAGE to separate unphosphorylated and phosphorylated proteins. These gels contain an immobilized ligand in complex with a divalent metal cation. The negatively charged phosphoryl group specifically interacts with the complex, leading to slower migration of phosphorylated protein compared to unphosphorylated protein. Eventually, the difference in relative mobility can be used to separate the two species (Kinoshita et al., 2009). Since the ligand also influences general gel migration behavior, no molecular weight estimation is feasible (Kinoshita & Kinoshita‐Kikuta, 2010). However, we confirmed the masses of our proteins using intact mass measurements (Table S2) and, hence, confirmed that *La*RldC and *Ro*RldC can be successfully phosphorylated using ammonium hydrogen phosphoramidate (Figure 4a). However, the degree of phosphorylation varies among homologs. For *La*RldC, phosphorylation appears to be pH‐dependent, with a more neutral pH increasing the amount of phosphorylated protein. *Ro*RldC was only partially phosphorylated under the tested conditions, while *Cp*RldC is not readily phosphorylated. Intrestingly, *Cp*RldCs lacks an otherwise conserved aspartate residue, which is substituted by a lysine in position 16. Previous studies have shown that similar substitutions can reduce phosphorylation efficiency of Rec domains (Bourret et al., 2021). This might explain why even extended incubation times allowed detection of little phosphorylated protein during intact mass measurements (Figure S10) and the only faint, shifted bands observed at pH 8 in Figure 4a. Under the tested conditions, illumination also did not increase the level of phosphorylated *Cp*RldC, a property shared with *Ro*RldC. However, for *La*RldC, we observed a noticeable increase in phosphorylated species in the presence of blue light, supporting a functional connection between the two sensory modules.

Since the Rec domain precedes the LOV domain, we also addressed the effect of phosphorylation on the photocycle. Indeed, we observed pronounced changes in the recovery kinetics of *La*RldC (Figure 4b). Phosphorylation of *La*RldC effectively increases the protein's recovery rate constant. While the unphosphorylated protein barely reverts to the dark state, phosphorylation drastically increases the recovery kinetics. This could be partially attributed to changes in ionic strength and the presence of additional ions resulting from the dissolved ammonium hydrogen phosphoramidate, which is necessary to maintain high levels of phosphorylation. However, control experiments with an unphosphorylatable D61A variant of *La*RldC showed that the effect on the recovery rate is mainly due to phosphorylation. For *Ro*RldC, we also observed an increased recovery rate constant, but to a lesser extent, potentially due to a lower fraction of phosphorylated protein and due to changes in ionic strength. Interestingly, the kinetics of photoproduct formation of *La*RldC were not affected by protein phosphorylation (Figure S11). Moving forward to the characterization of kinetic properties, there is still one member of each LOV‐GGDEF linker length group available for in vitro diguanylate cyclase activity assays.

### 2.5 In vitro activity

**FIGURE 5:**
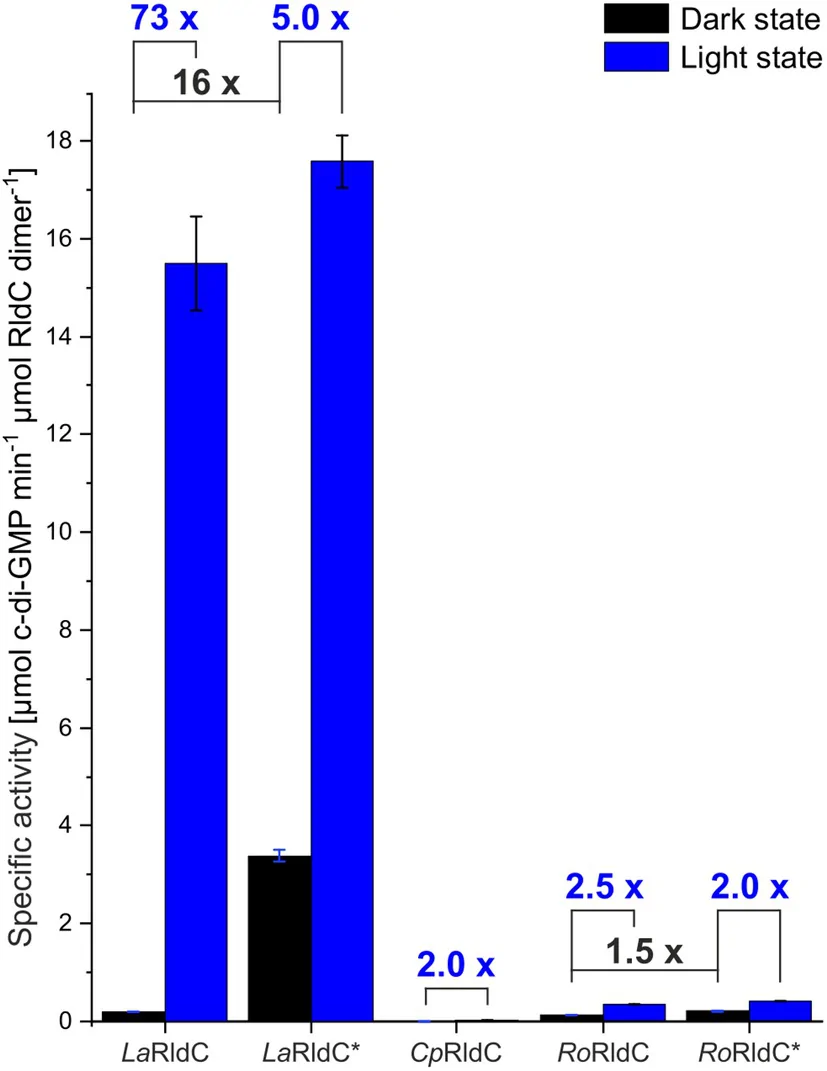
Specific activity of RldC homologs. Values for 100 μM substrate concentration are shown. Dark state activities are depicted in black and light state activities in blue. * Indicates phosphorylated protein. Fold changes between states are indicated above the bars. The SE of the linear regression estimate is shown as an error bar.

To investigate the in vitro activity of *La*RldC, *Cp*RldC, and *Ro*RldC, we measured initial velocities (*v*_0_) of the homologs using an offline HPLC‐based assay. For initial comparisons, all in vitro activities were determined at a GTP concentration of 100 μM (Figure 5), since other GGDEF‐proteins have typically been characterized using GTP concentrations between 50 and 500 μM (Vide et al., 2026; Tran et al., 2024; Gourinchas et al., 2017; Teixeira et al., 2021; Vide et al., 2023). To avoid any impact of high PA concentrations on activity, we diluted the samples directly before starting the reaction, thereby reducing the PA concentration from 50 to 5 mM. All homologs showed some degree of light activation, but *La*RldC showed the biggest change with a 73‐fold increase. Due to the high activity of the protein, we could only use the 5 and 10 s timepoints to determine *v*_0_. Reducing the enzyme concentration further was not an option, as concentrations below 1 μM decreased specific activity, most likely due to a monomer‐dimer equilibrium (Figure S12). *Cp*RldC and *Ro*RldC exhibited smaller changes of 2‐fold and 2.5‐fold, respectively. In line with the in vivo screening, *Cp*RldC had a significantly lower specific activity than the other two homologs. In addition, the conversion of GTP was accompanied by the formation of the reaction intermediate 5′‐pppGpG, suggesting suboptimal alignment of the GGDEF protomers (Böhm et al., 2021). Since phosphorylation of *Cp*RldC was shown to be inefficient, only *La*RldC and *Ro*RldC were characterized regarding their response to the second input signal. Phosphorylation of dark state *La*RldC increased activity 16‐fold; shifting the protein to the light state further increased activity 5‐fold. On the other hand, *Ro*RldC increased activity upon phosphorylation in the dark state only by 1.5‐fold and a further 2.0‐fold increase when illuminated. These results show that the individual signals and the combination of both can modulate DGC activity. However, the extent of the regulation differs substantially between homologs.

## 3 DISCUSSION

Detailed studies of dual sensor photoreceptors remain scarce, most likely due to the complex nature of these proteins and their frequently unknown physiological roles (Kobayashi et al., 2023; Shin et al., 2019). Here, we characterized several dual sensor diguanylate cyclase homologs and investigated their responses to both illumination and phosphorylation.

The initial in vivo screening enabled qualitative analysis of the enzymatic activity of all selected homologs under both dark and illuminated conditions. *Cp*RldC appeared inactive on the plate screen (Figure 1d) and was later shown to exhibit very low specific activity, yet measurable light regulation in our in vitro measurements. Since we investigated multiple homologs, we tried to maintain comparable buffer conditions throughout our analyses. Yet, for some proteins, these conditions might not be optimal, and additional studies could be performed to improve activity by varying pH, temperature, and/or ionic strength (Zhou & Pang, 2018). The variants of *La*RldC exchanging the phosphorylated aspartate for alanine (D61A) and the adduct‐forming cysteine for serine (C207S) were used in the in vivo assay as additional controls. For one, we checked for any cross‐talk of histidine kinases present in *E. coli* Bl21 (DE3) with the Rec domain. On the other hand, it helped to confirm that the sometimes‐subtle differences between dark and illuminated plates are indeed due to light‐induced changes in enzymatic activity. Additionally, the D61E variant was tested to see whether this amino acid exchange could mimic the phosphorylated state (Klose et al., 1993; Kurepa et al., n.d.). While the colonies slightly differed from those of the wild‐type and the D61A variant, the phosphomimetic D61E is not as efficient in stimulating DGC activity as illumination. This is in line with the effect of phosphorylation or illumination in enzymatic activity assays. While we identified several homologs that showed light regulation in vivo, not all could be purified due to protein solubility issues. Interestingly, due to their high DGC activity, some homologs were copurified with c‐di‐GMP, most likely bound to the I‐site of the GGDEF domain (Christen et al., 2006), which is conserved in all investigated homologs. When we performed native MS measurements to confirm cofactor loading and the dimeric state of all homologs, however, we did not observe evidence of bound c‐di‐GMP. This suggests that the interaction between product and protein is not strong enough to withstand ionization or is lost during the exchange from storage buffer to ammonium acetate. The feedback inhibition via the I‐site also complicates the interpretation of the in vivo screening, since it affects the homologs to different degrees.

To mitigate such drawbacks, we proceeded to in vitro assays and used RocR (Rao et al., 2008) as an established EAL phosphodiesterase to successfully remove c‐di‐GMP from *La*RldC and *Ro*RldC. Interestingly, product inhibition at the I‐site substantially influences conformational dynamics, as in the case of *La*RldC, a disulfide bond between the two protomers formed rapidly after c‐di‐GMP removal. Disulfide bond formation is likely due to a cysteine residue in the *A*′*α* helix of the LOV domain. The residue is part of the coiled‐coil linker connecting the Rec and LOV domains, which influences communication between the two. In fact, this suggests additional regulation of the cross‐talk between the LOV and the Rec domains, along their tentative signal integration pathway, with intracellular redox state. We only observed this disulfide bond in *La*RldC and *Ls*RldC, where this specific cysteine is conserved. Structure predictions of the dimers also showed the close proximity of these cysteines. During method development for *La*RldC kinetics, it was observed that oxidized samples had a substantially reduced turnover and lower fold‐changes of light activation. Potentially, cysteine oxidation could serve as an additional input on RldC regulation as observed in other redox‐sensing systems (Cremers & Jakob, 2013; Martins et al., 2026; Li et al., 2022). It was also reported that cyanobacteria exhibit a certain light/dark modulation of thiol oxidation (Guo et al., 2014). Therefore, the additional influence of light on pH and redox might even result in extended multimodal regulation regimes. As pH alters protonation states of catalytic residues and metal coordination at the Rec and GGDEF domains, it will further influence RldC activity; along this line, cellular redox also influences the LOV cofactor redox state and/or cysteine thiol chemistry. Eventually, all of these mechanisms will be used to tune allosteric communication. Thus, Rec phosphorylation and LOV photoactivation operate within a broader, time‐varying chemical environment that controls GGDEF activation. While these findings open new questions regarding output regulation, we did not further investigate redox effects at this time and instead focused on illumination and phosphorylation.

To address the effects of illumination, we analyzed the spectroscopic properties of all purified RldC homologs. We identified three representatives that did not exhibit the characteristic LOV photoproduct with an absorption maximum at 390 nm. Instead, the absorption maximum was blue‐shifted toward 370 nm (Figure S13A). This unusual behavior coincided with the necessity to use a 470 nm LED for maximum photoconversion, which was especially apparent for *La*RldC and *Ls*RldC. While influences of light quality have been reported for LOV domains before (Losi & Gärtner, 2016), it is intriguing to observe such a clear preference for higher wavelengths in terms of activation, despite the dark state spectrum not being red‐shifted itself (Figure S13B). Previous studies have shown that ZEITLUPE (ZTL) from *Arabidopsis* also shows a blue‐shift of the light‐state spectrum compared to other LOV proteins (Zoltowski & Imaizumi, 2014). Interestingly, there appears to be some correlation with a slow thermal reversion of the photoadduct in LOV domains and these peculiar spectral properties. ZTL and *Ro*RldC actually exhibit similarly slow dark state reversions. The even slower recovering *La*/*Ls*RldCs feature an even stronger blue shift of the flavin cysteinyl adduct spectrum. However, there are clearly also other factors influencing dark state recoveries, as very slow LOV proteins, like for example FKF1, can also feature characteristic LOV‐adduct spectra (Pudasaini et al., 2017; Pudasaini & Zoltowski, 2013). Spectral tuning of LOV domains is considered interesting for multiple applications, including optogenetics. While this usually focuses on changing the properties of the dark state, not the light state, using lower‐energy, and therefore less damaging, radiation for activation could be beneficial. We tried to identify the specific residues responsible for the shifted light‐state spectrum by examining the cofactor's surroundings. Therefore, we used the dark‐adapted and light‐adapted structures of AsLOV2 (Dietler et al., 2022) to identify residues and compared them with those of our purified homologs and ZTL. While some differences were observed for *Cp*RldC near the flavin dimethyl‐benzyl ring, this homolog's spectrum showed no atypical characteristics. For those homologs with a clearly shifted spectrum, like *La*RldC, no obviously deviating amino acids could be identified, suggesting that second shell residues exert this intriguing influence.

To confirm that we indeed observe photoadduct formation, we also characterized a C207S variant of *La*RldC. While the dark state spectrum of the protein changed due to the exchange, similar amino acid substitutions have already been shown to alter the absorption spectrum of other LOV proteins (Kasahara et al., 2010; Salomon et al., 2000). Generally speaking, the flavins' short wavelength peak depends more on solvent polarity and hydrogen bond formation, and can therefore be influenced more readily (Losi & Gärtner, 2016). Without the adduct‐forming cysteine, we no longer observed a LOV‐characteristic response to blue light. Photoreduction of the variant under anaerobic conditions also yielded a qualitatively different spectral change compared to the wild type. Together, these findings support photoadduct formation in all our systems and hint at subtle differences in the local flavin environment influencing the electronic properties of the flavin. Additionally, once exposed to oxygen, the reduced FMN reoxidized swiftly, in contrast to the slow recovery of the very stable light state observed for *La*RldC. These characteristics hint at the protein's physiological role. Studies of photoreceptors in *Arabidopsis thaliana* have shown that LOV domains with significantly different recovery rates, namely ZTL and FKF1, are associated with specific functions. FKF1, which slowly reverts to the ground state, operates in a day‐loop, whereas the fast‐recovering ZTL senses light–dark transitions (Pudasaini & Zoltowski, 2013). While a very stable light state might not be desirable for all optogenetic applications, such a behavior could prove interesting, as illumination with near‐UV light can efficiently shift the protein back to its dark state, thereby eliminating the need for constant illumination while retaining light control. In contrast to what was shown for LOV2 of *Adiantum*, this process is not faster than activation but rather occurs on a similar timescale (Papin et al., 2005).

To test the effect of phosphorylation, we used the small‐molecule phosphodonor phosphoramidate, which features a nitrogen‐phosphate bond similar to that of natural activated histidine kinases. The compound, in fact, was able to phosphorylate several RldCs. However, depending on the homolog, the degree of phosphorylation varied substantially and was further influenced by the phosphorylation conditions. In the case of *La*RldC, we found that a lower pH led to more complete phosphorylation. Similar observations were made for a Rec domain that is part of a virulence switch in *Salmonella*. There, pH dependence was attributed to a histidine residue proximal to the phosphorylated aspartate (Shetty & Kenney, 2023). A sequence alignment revealed that this histidine residue is also present in *La*RldC, and structure predictions indicate its proximity to Asp61. *Cp*RldC and *Ro*RldC do not feature this histidine residue and did not exhibit a clear pH dependence. In addition to the pH dependence of *La*RldC, we observed that illumination (Figure 4a) increases the amount of phosphorylated protein. This could be caused by light‐induced changes at the dimer interface, including the *A*′*α* helices, that favor or stabilize the phosphorylated state. Since signal transduction from the Rec domain via the LOV domain to the DGC will depend on the domain‐connecting coiled‐coil linkers, such an allosteric cross‐talk of the individual sensors should be an integral part of the molecular signaling mechanism. In fact, the influence of substituting the phosphorylatable Asp in the Rec domain (D61A variant) on thermal recovery of the LOV domain further highlights such an allosteric communication pathway between domains; a feature frequently demonstrated for sensory proteins using methods to address their conformational dynamics in various functional states (Swingle et al., 2025; Dikiy et al., 2019; Heintz & Schlichting, 2016; Lindner et al., 2017; Watad et al., 2025; Winkler et al., 2013, 2014).

In the case of *Cp*RldC, neither Phos‐Tag™ SDS‐PAGE nor intact MS measurements showed substantial amounts of the phosphorylated species. While the molecular origin for such behavior can be manifold, one interesting difference to the other homologs is the evolutionary loss of one of the Mg^2+^‐coordinating residues in its Rec domain. In fact, a positively charged Lys residue replaces the negative charge of Asp 16 in *Cp*RldC and could explain the difficulties in phosphorylation via small molecule phosphodonors. Studies investigating CheY, a stand‐alone Rec domain, have shown that an analogous D13K variant is less efficiently phosphorylated by its cognate kinase CheA (Bourret et al., 2021). It should be noted that this variant of CheY exhibits similar behavior to the phosphorylated wild type and can therefore be considered a constitutively active variant. However, structural studies showed that CheY wild type and D13K variant adopt the same structure when crystallized (Jiang et al., 1995). Therefore, we did not expect *Cp*RldC to exhibit a similar behavior, as the proposed activation mechanism would involve structural rearrangements transmitted through the alpha‐helical linkers rather than protein–protein interactions.

Similar to the phosphorylation experiment using Phos‐Tag™ gels, preliminary kinetic measurements assessed the effect of pH on enzymatic activity. We observed an influence of buffer pH on the activity and fold changes of the studied homologs. Interestingly, measurements at pH 7.0 yielded lower fold changes, whereas measurements at pH 8.0 yielded higher fold changes. While this contrasts the observed phosphorylation efficiency via Phos‐Tag™ analysis, we chose to use pH 8.0 for a more detailed kinetic characterization; also, because cyanobacteria have been shown to have a higher cytosolic pH in light, which assists carbon fixation. Measurements in *Synechococcus elongatus* show a cytosolic pH of ≈7.3 in dark‐acclimated cells, increasing by 1.1 units in the light (Mangan et al., 2016). Since the in vivo screening reflects the activity and fold changes at the physiological pH of *E. coli* cytosol, it also explains why the in vitro fold change*s* of *La*RldC are much higher than indicated by the screening. The in vitro measurements showed that phosphorylation of the N‐terminal Rec domain increases activity by 16‐fold, while illumination results in a 73‐fold change. Although both individual signals and their combination led to higher activity and hence reflect an OR logic, this does not describe the protein's behavior in every detail. Phosphorylation of the Rec domain has less impact than illumination, suggesting that the organism primarily responds to light to upregulate c‐di‐GMP production and that the signal that triggers phosphorylation has less impact. This provides the opportunity to fine‐tune cellular responses in response to external factors. The observed influence of phosphorylation on dark state recovery and the increased phosphorylation ratio in the light‐state add further complexity. These observations highlight the allosteric nature of both signals. While we were able to characterize the effect of phosphorylation for this homolog in vitro, it is unclear which external factors cause phosphorylation. The gene preceding *La*RldC encodes a complex multi‐domain hybrid histidine kinase that features a periplasmic Cache domain (dCache_1, PF02743), followed by a transmembrane segment and at least four cytosolic domains—PAS, HAMP, HK, and Rec. While Cache domains are the most abundant extracellular sensor domains in prokaryotes, their ligands are diverse and still remain largely elusive (Upadhyay et al., 2016; Matilla et al., 2022). While it is likely that this hybrid kinase would phosphorylate *La*RldC in vivo, we cannot predict which ligand controls its activity.

At the level of RldC control, one of our initial aims was to determine whether variation in the linker length between the LOV and GGDEF domains exerts a similarly pronounced effect on dark‐to‐light activity changes as previously reported for LOV‐GGDEF proteins (Vide et al., 2023). However, the limited number of RldCs that could be fully characterized prevented us from identifying clear general trends. The multidomain architecture of RldCs, comprising three functional domains connected by two linker regions, provides numerous structural and functional features that may influence enzymatic output. Consistent with this, pairwise sequence identity varied substantially between linker‐length groups (Table S4). More importantly, domain‐specific sequence comparisons (Table S5) further revealed that the linker regions exhibit greater variability than the core domains. From an evolutionary perspective, this makes sense, since linker regions act as evolutionary hotspots for functional tuning, as even single amino acid substitutions can substantially alter enzymatic activity and signal transduction (Vide et al., 2026). Intrestingly, the linker connecting the Rec and LOV domains is the least conserved region across the different groups. While sequence diversity and length differences in this region (Figure S1) have not been probed systematically in this study, the importance of related linker regions has also been pointed out in Rec‐GGDEF systems (Teixeira et al., 2021). In addition, domain specific comparisons also highlighted the different degrees of conservation of individual domains. *Cp*RldC is a point‐in‐case where LOV and GGDEF domains show comparable sequence identities within the LOV‐GGDEF linker length group, but the Rec domain features substantially lower identities. Apparently, also horizontal gene transfer and domain shuffling together with system specific microevolution cannot be ignored—further complicating the ability to draw general conclusions from a limited set of RldC homologs.

Nevertheless, *La*RldC proved to be a promising system that allowed us to quantify changes in activity caused by illumination and phosphorylation via our biochemical characterization. Still, the molecular mechanism of this regulation remains elusive at this point. While there are multiple regulation and signal transduction mechanisms published concerning LOV domains (Swingle et al., 2025; Chaudhari et al., 2025; Herrou & Crosson, 2011; Vide et al., 2023), the combination of a Rec domain linked to a LOV domain has not been described before. It appears intriguing to consider a potential Rec‐signal feeding into the *A*′*α* helix of LOV, a proven central element of LOV signaling (Möglich et al., 2010). Eventually, this will affect the LOV‐GGDEF linker in ways similar to LOV‐, phytochrome‐ or Rec‐GGDEF couples (Vide et al., 2026; Gourinchas et al., 2018; Teixeira et al., 2021). To address some of the open questions, we are currently following up on an integrative structural biology approach combining data from x‐ray crystallography, *in crystallo* optical spectroscopy and hydrogen deuterium exchange coupled to mass spectrometry analyses to probe in solution conformational dynamics across multiple functional states.

In conclusion, *La*RldC was identified as a particularly interesting dual sensor diguanylate cyclase, showing activity modulation by both phosphorylation and illumination. Based on these results, the protein can be described as operating with a molecular OR logic. Additional factors, such as pH variations and redox inputs, which are certainly encountered in an in vivo context, can also affect activity, rendering this system a peculiar, multilayered enzyme evolved by nature.

## 4 MATERIALS AND METHODS

### 4.1 Gene selection and cloning

InterPro and PSI‐BLAST were used to identify sequences with a Rec‐LOV‐GGDEF architecture. Multiple sequence alignments were filtered using conserved residues and motifs of the domains as constraints. We classified groups based on the linker between the LOV and GGDEF domains. Similar to previously characterized LOV‐GGDEF homologs (Vide et al., 2023), we defined three classes with different linker lengths, differing by seven or 14 residues.

Genes were ordered from Thermo Fisher Scientific and sequences codon‐optimized for expression in *E. coli*. To assemble the plasmids, we performed a Gibson assembly to clone the genes into a pET‐M11 vector with an N‐terminal His‐tag and a TEV protease cleavage site using the NEBuilder HiFi DNA Assembly Cloning Kit.

### 4.2 In vivo screening

Plasmids encoding the RldC homologs were transformed into *E. coli* BL21 (DE3) and plated on LB Kan plates. After overnight incubation at 37°C, multiple colonies were used to inoculate a 3 mL liquid culture. The culture was grown at 37°C to an OD_600_ of at least 0.5. A volume of 0.5 mL with an OD_600_ of 0.5 was centrifuged briefly to pellet the cells. The supernatant was discarded, and the cells were resuspended in 25 μL LB medium. Under non‐actinic conditions, 2 μL of the resuspended cells were spotted on a Congo Red screening plate (LB (Luria/Miller); 60 mg/L Congo red; 25 mg/L Coomassie G‐250; 20 g/L Agar). Plates were wrapped in thin foil and kept dark or illuminated using an LED light panel (Lunartec). The screening plates were incubated at 20°C for at least 12 h. Afterward, plates were kept at 4°C until pictures were taken with a Canon EOS 1100D, typically after 3–4 days.

### 4.3 Protein production and purification

To produce the various RldC homologs, we transformed the plasmids described above into *E. coli* BL21 (DE3). The preculture was grown to an OD_600_ of 1. The main cultures were inoculated with an OD_600_ of 0.02 and subsequently grown to an OD_600_ of 0.5–0.6. Afterwards, the cultures were transferred to 16°C, and after 45 min, IPTG was added to a final concentration of 0.1 mM. Cells were harvested after approximately 18 h (15 min, 4°C, 3850 × *g*) and stored at −20°C until purification.

Cell pellets were resuspended in 5 mL lysis buffer per gram wet cell weight (*Ap*, *Cp*, and *La*: 50 mM HEPES pH 7.0, 500 mM NaCl, 2 mM MgCl_2_, 30 mM Imidazole; *Ls* and *Ro*: 50 mM CHES pH 9.0, 300 mM NaCl, 2 mM MgCl_2_, 30 mM Imidazole). Lysozyme (0.5 mg/mL), Protease Inhibitor Cocktail (Roche cOmplete™, EDTA free) and DNase I (Roche, grade II) were added to assist cell disruption. After 40–60 min of resuspending at room temperature, the cells were disrupted using a B. Braun Labsonic U sonicator (3 × 5 min). The suspension was centrifuged (45 min, 4°C, 37,000 × *g*) to obtain the supernatant. The supernatant was carefully removed and loaded onto a gravity flow Ni‐NTA column (Macherey‐Nagel Protino, 35 mL, packed with Cytiva Ni Sepharose 6 Fast). 3 CVs of lysis buffer supplemented with 100 μM FMN (Panreac, AppliChem) were used to ensure even cofactor loading. Then, 6 CVs of lysis buffer containing 40 mM imidazole were used as wash buffer. The protein was eluted using lysis buffer with 250 mM imidazole. Overnight dialysis, lowering the imidazole concentration to 45 mM, was coupled with tag removal using TEV protease (ratio 1:15 TEV:target protein). The protein solution was then reloaded onto a Ni‐NTA column to remove the His‐tagged TEV protease and the cleaved tag. The flow‐through was concentrated using an Amicon (Merck, 30 kDa MWCO, 3000 × *g*, 4°C), followed by size exclusion chromatography as a polishing step (Cytiva, S200 increase 10/300 GL).

To remove bound c‐di‐GMP, the phosphodiesterase RocR, purified according to Rao et al. (2008), was used. Estimating the protein‐to‐c‐di‐GMP ratio to be around 1:1, we mixed RocR and the RldC homolog at a 1:5 ratio. To increase turnover, the reaction buffer was supplemented with 100 mM MgCl_2_. Complete removal was confirmed via HPLC analysis of a denatured reaction mixture sample (conditions similar to the kinetic measurements described below). Afterwards, RocR was separated via Ni‐NTA affinity chromatography. For *La*RldC, the addition of 1 mM TCEP during and after c‐di‐GMP removal was necessary to prevent intermolecular disulfide formation.

### 4.4 Native mass measurements

The native MS analysis method used in our lab is described in Li et al. (2026). Briefly, 50 pmol of protein samples (5 μL at 10 μM concentration) were desalted on an AdvanceBio SEC 300 Å (2.7 μm, 4.6 × 50 mm, Agilent) size‐exclusion HPLC‐column equilibrated in 150 mM ammonium acetate, pH 7. A Shimadzu Nexera UHPLC system was used for automated injections and desalting via the column at a flow rate of 0.2 mL/min. The eluting proteins were directly injected into a Bruker maxis II ultra‐high resolution Q‐TOF device and the elution volumes containing salts were diverted to the waste valve. Mass spectra were acquired in an *m*/*z* range between 500 and 10,000 with 2.5 bar nebulizer pressure and 8 L/min dry gas setting. The source temperature was set to 325°C and isCID energy to 100 eV. Transfer time and pre‐pulse storage were optimized for nMS with 180 and 40 μs, respectively. Calibration of the mass spectrometer in the *m*/*z* range from 1822 to 5460 was performed using the ESI‐tune mix at elevated concentrations, allowing assignments of dimeric and trimeric species. Data were analyzed using DataAnalysis (Bruker) and maximum entropy deconvolution of the protein signals.

### 4.5 Phosphorylation and intact mass measurements

Ammonium hydrogen phosphoramidate was synthesized as previously described (Cotton, 1971). For protein phosphorylation, samples at a concentration of 10 μM were incubated with 100 mM ammonium hydrogen phosphoramidate for specified times at 20°C. The phosphodonor specifically targets the aspartate residue of the receiver domain, which would be phosphorylated by a cognate kinase in vivo. Until measurement, the samples were kept at 4°C. Intact mass measurements were performed in‐house as previously described by Tran et al. (2024) on a Bruker Impact II QTOF mass spectrometer. Briefly, 5 μL samples were desalted on a Shim‐pack Scepter C4‐300 (G) column (3 μM) by washing with 1% ACN in the presence of 0.1% formic acid. A gradient from 1% to 95% ACN was used to elute the proteins. Integration and maximum entropy deconvolution were performed in Bruker “DataAnalysis.”

### 4.6 Phos‐Tag gels

Phos‐Tag™ acrylamide was purchased from FUJIFILM Wako Pure Chemical Corporation and used to prepare Zn^2+^‐Phos‐Tag™ SDS‐Gels with 6% acrylamide. Similar to intact mass measurements, proteins were incubated with PA at 10 μM protein concentration. After 1 h of incubation, reactions were stopped by adding 3× sample buffer (according to Wakos Phos‐Tag™ Guidebook, but without reducing agent). Samples were loaded without the usual heating denaturation and run at 20°C with constant voltage (100 V).

### 4.7 Spectroscopic measurements

Spectroscopic measurements were performed using a Specord 200 PLUS dual‐beam UV–vis spectrophotometer (Analytik Jena). Proteins were diluted to 5 μM with storage buffer (Table S2). The first dark spectrum was recorded under non‐actinic conditions. To shift proteins into the light state, a ThorLabs 455 or 470 nm mounted LED (3 mW/cm^2^) was used. Samples were illuminated for specified times, after which spectra were measured. Spectra were recorded until no further light‐induced change was observed. Dark state recovery was determined by collecting spectra from 300 to 650 nm at predetermined time intervals to detect potential aggregation effects during long measurement times.

### 4.8 Kinetic measurements

Protein stocks were diluted with reaction buffer (100 mM Tris pH 8.0, 500 mM NaCl, 10 mM MgCl_2_, 1 mM TCEP) to 2 μM. Dark state samples were started by the addition of GTP. For the individual time points, 40 μL of the reaction was withdrawn and stopped by the addition of EDTA and additional heat denaturation (96°C, 1 min). For light state measurements, samples were pre‐illuminated for time intervals based on the initial spectroscopic characterization (*La*RldC: 5 min; *Cp*RldC: 90 s; *Ro*RldC: 90 s), before adding GTP to start the reaction. Samples were centrifuged for 10 min at 13,000 rpm before HPLC analysis. The samples were separated isocratically (10 mM K_2_HPO_4_; 1 mM EDTA) on a C18 column (Bischoff ProntoSIL, 1250‐5‐C18 ace‐EPS, 100 × 4.6 mm) at 25°C. Nucleotides were detected based on 254 nm absorbance, with quantification based on relative peak heights. All measurements were performed in triplicate to evaluate the standard deviation.

## AUTHOR CONTRIBUTIONS

**Maximilian Fuchs:** Investigation; writing – original draft; writing – review and editing; data curation; formal analysis; methodology; visualization. **Andreas Winkler:** Conceptualization; funding acquisition; writing – review and editing; project administration; resources; supervision; data curation; validation; writing – original draft.

## FUNDING INFORMATION

M. F. acknowledges support as an associate student from the Austrian Science Fund (FWF) grant DOC‐130. This research was funded in whole or in part by the Austrian Science Fund (FWF) [grant doi: 10.55776/P34387; to A. W.]. For open access purposes, the author has applied a CC BY public copyright license to any author accepted manuscript version arising from this submission.

## CONFLICT OF INTEREST STATEMENT

The authors declare that they have no conflicts of interest.

## Supporting information


**Figure S1:** Sequence alignment of selected RldC homologs. The alignment was filtered for redundancy, eliminating sequences with identities larger than 95%. Individual domains and linker regions are labeled. Colors correspond to the percentage identity as shown in the bottom‐right corner.
**Figure S2:** SDS‐PAGE analysis of individual homologs. Gels were used to judge the solubility of individual homologs after overnight protein production. p.I = before induction; H = harvested cells after overnight protein production; P = cell pellet after extraction of soluble fraction; Sn = supernatant after centrifugation containing soluble fractions.
**Figure S3:** Absorption spectra identifying bound c‐di‐GMP. (A) UV absorbance differs between CpRldC (no bound c di‐GMP) and LaRldC (bound c‐di‐GMP). (B) Comparison of LaRldC before and after removing bound c‐di‐GMP. The difference spectrum matches the spectrum of guanosine.
**Figure S4:** HPLC traces and peak spectra confirming bound or converted c‐di‐GMP. (A) Denatured sample of LaRldC showing bound c‐di‐GMP. (B) Absorption spectrum of the peak visible in panel (A). (C) Denatured sample of LaRldC after incubation with RocR showing conversion of c‐di‐GMP to pGpG. (D) Absorption spectrum of the pGpG peak shown in (C).
**Figure S5:** FPLC traces of the RoRldC purification. The elution volume of the main peak changes once bound c‐di GMP is removed. Purification on a Cytiva S200 increase 10/300 column equilibrated in 10 mM Tris pH 8.0, 500 mM NaCl, 2 mM NaCl.
**Figure S6:** Intact Mass measurements of LaRldC and LsRldC. (A) LaRldC measured after removal of bound c‐di‐GMP. In addition to the monomer mass, a dimer is also present. The expected mass for the protein is 53,915.8 Da. (B) Measurement of LaRldC after the addition of TCEP to the buffer and detection of only the monomer's mass. The minor amount of dimer can be considered an artifact of maximum entropy deconvolution. (C) LsRldC after protein purification. Monomer and dimer peaks are visible. Expected monomer mass of 57,230.4 Da. (D) Intact mass measurement of LsRldC after addition of TCEP to the buffer. Only the peak corresponding to the monomer remains.
**Figure S7:** Influence of light quality on dark‐ to light‐state transition of LaRldC. (A) Sample illuminated with 455 nm blue light LED (ThorLabs, 3 mW/cm^2^) for indicated amounts of time. (B) Illumination with 470 nm blue light LED (ThorLabs, 3 mW/cm^2^) for specified times.
**Figure S8:** Single exponential fits of thermal recoveries. (A) Shows ApRldC recovery data (black) and exponential fit (red). Equation with rounded parameters *y* = −0.06 * *e*(−*x*/150) + 0.09; τ (mean lifetime) = 150 min. (B) Recovery data of CpRldC in black with calculated fit in red. *y* = −0.10 * *e*(−*x*/101) + 0.11; τ = 101 min. (C) Measured recovery data of RoRldC (black) with exponential fit (red). y = −0.09 * *e*(−*x*/253) + 0.1; τ = 253 min.
**Figure S9:** UV illumination of LaRldC. Illumination with 395 nm light (2 mW/cm^2^) switches the protein back to the dark state. Times of illumination are specified in the legend.
**Figure S10:** Intact mass measurement after incubation with 50 mM phosphoramidate. (A) CpRldC after multiple hours of incubation at 20°C. Only moderate amounts of phosphorylated protein were detected. The approximately 80 Da mass difference is characteristic of protein phosphorylation. (B) Mass spectrum showing phosphorylation of LaRldC. The majority of the protein was phosphorylated after 1 h incubation at 20°C. The peak at −18.0 Da is due to the neutral loss of H_3_PO_4_ from the phosphorylated species, a well‐documented phenomenon in phosphorylated proteins (Cotton, 1971; Li et al., 2026).
**Figure S11:** Activation of LaRldC with and without phosphorylation. Shows the decrease of the 450 nm absorbance in response to illumination with 470 nm blue light. The phosphorylated sample indicated by * was incubated with 50 mM phosphoramidate before the measurement.
**Figure S12:** Concentration dependence of specific activity for LaRldC. The data indicates that lower concentrations than 1 μM show lower specific activity. This suggests the possibility of a monomer‐dimer equilibrium influencing the protein's activity. Concentrations above 2 μM are affected by high substrate turnover, thereby limiting the achievable specific activity.
**Figure S13:** Differences in dark‐ and light‐state absorption spectra of the studied RldC homologs. (A) The light state of LaRldC and LsRldC is significantly blue‐shifted compared to the usual 390 nm maximum observed in other LOV domains. Spectra normalized based on the observed maximum. (B) Normalized dark‐state spectra of all soluble homologs, comparing the maximum of the UV‐A band.
**Figure S14:** Uncut image of the in vivo screening plates shown in Figure 1.
**Figure S15:** Complete Phos‐Tag™ SDS‐PAGE shown in Figure 5. The colored protein standard was used to follow the progress of the gel once the running front left the gel. Due to the gel composition, the protein standard cannot be used to estimate molecular masses.
**Table S1:** Identifiers and background information on RldC homologs. Protein acronyms are built by adding the first letter of the genus and species to the RldC (Receiver LOV‐activated diguanylate cyclase) abbreviation and were given to homologs selected for the study. Shades of gray highlight the proposed classification based on different linker lengths between the LOV and GGDEF domains.
**Table S2:** Data obtained from mass spectrometry measurements. Expected masses were calculated using Prot pi (www.protpi.ch/Calculator/ProteinTool). Holo dimer masses contain the average masses of 2 molecules of FMN in addition to two protein protomers. The measurement accuracy was calculated in ppm. Shades of gray highlight the classification by LOV‐GGDEF linker length.
**Table S3:** Storage buffer composition for each of the studied homologs. Shades of gray highlight the classification by LOV‐GGDEF linker length.
**Table S4:** Percentage identity based on pairwise alignment of the studied homologs' amino acid sequences. Values are given in %. Shades of gray highlight the classification by LOV‐GGDEF linker lengths. Cell coloring was generated using Excel's three‐color conditional formatting scale, with fixed thresholds of 15%, 55%, and 95%.
**Table S5:** Percentage identity based on pairwise alignment of individual domains and functional elements. Domain and linker boundaries are visible in Figure S1. Values are given in %. Shades of gray highlight the classification by LOV‐GGDEF linker length. Cell coloring was generated using Excel's three‐color conditional formatting scale, with fixed thresholds of 15%, 55%, and 95%.

## Data Availability

The data that support the findings of this study are openly available in the TU Graz Repository at https://repository.tugraz.at, reference number https://doi.org/10.3217/d4xbt-ngy72.
