# Supplementary material for: Molecular logics in dual sensor regulation of diguanylate cyclase activity—Phosphorylation OR blue‐light activation
Source: Protein Sci. 2026 Aug 24;35(9):e70765. doi: 10.1002/pro.70765 (PMC13501900; doi:10.1002/pro.70765)
Supplement: Supplementary file 1 [file PRO-35-e70765-s001.pdf]

Molecular logics in dual sensor regulation of diguanylate cyclase activity - Phosphorylation OR blue-light activation

Maximilian Fuchs[1], Andreas Winkler[1,2,*]

Affiliations

[1] Institute of Biochemistry, Graz University of Technology, Graz, Austria

[2] BioTechMed-Graz, Graz, Austria

[*] Corresponding author. E-Mail: andreas.winkler@tugraz.at

Supplementary material

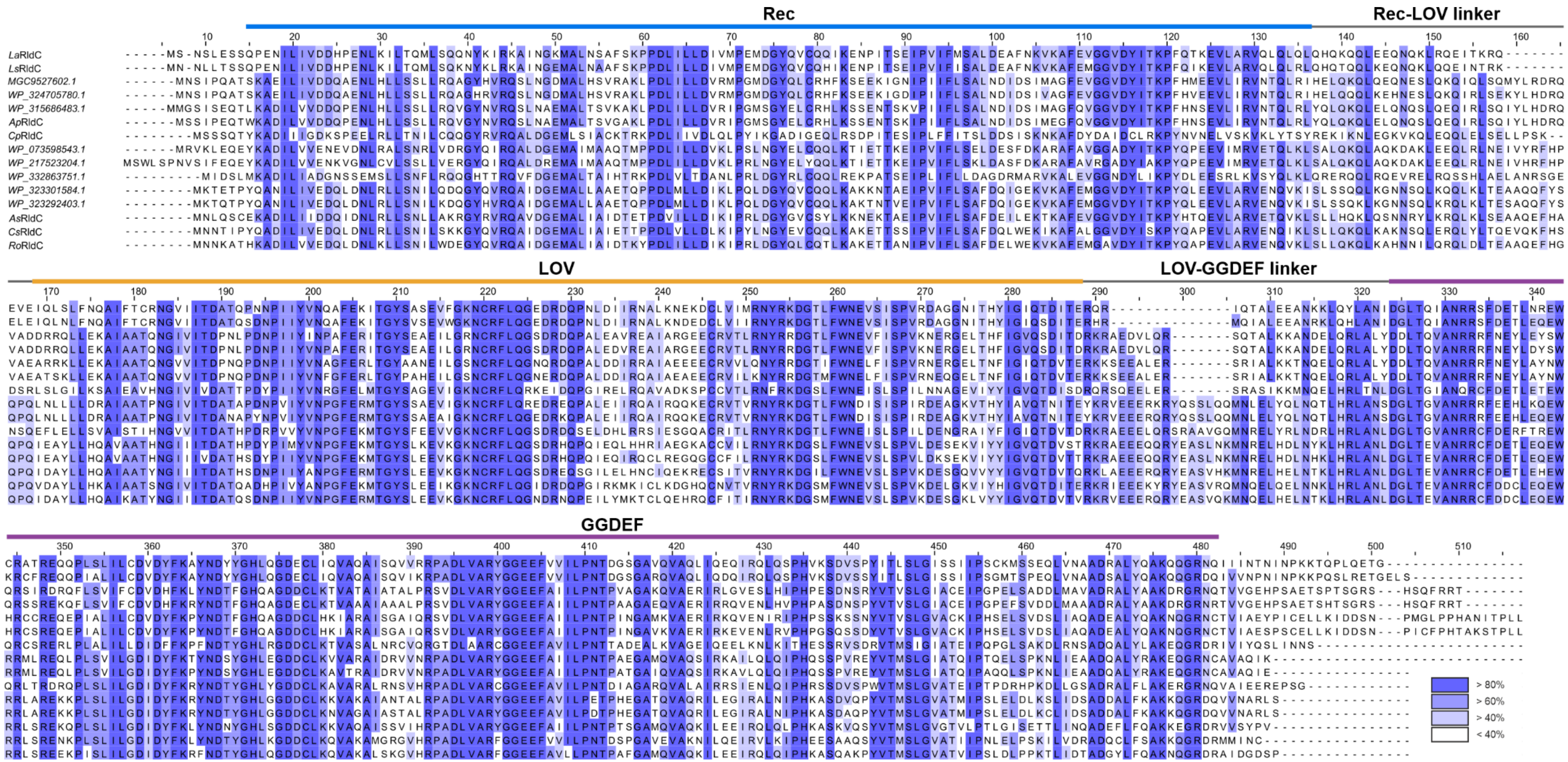

Figure S1: Sequence alignment of selected RldC homologs. The alignment was filtered for redundancy, eliminating sequences with identities larger than 95%. Individual domains and linker regions are labelled. Colours correspond to the percentage identity as shown in the bottom-right corner.

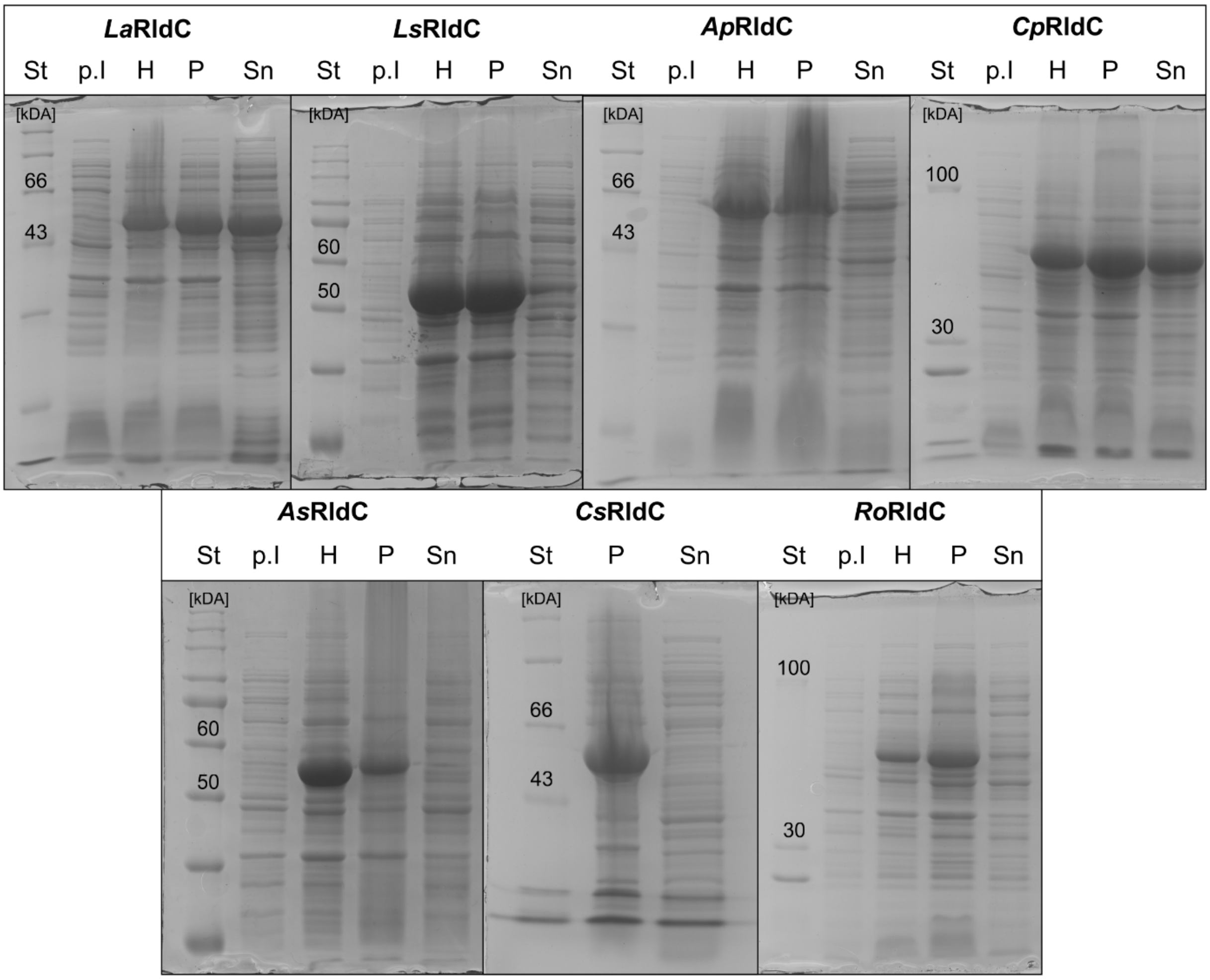

Figure S2: SDS-PAGE analysis of individual homologs. Gels were used to judge the solubility of individual homologs after overnight protein production. p.I = before induction; H = harvested cells after overnight protein production; P = cell pellet after extraction of soluble fraction; Sn = supernatant after centrifugation containing soluble fractions.

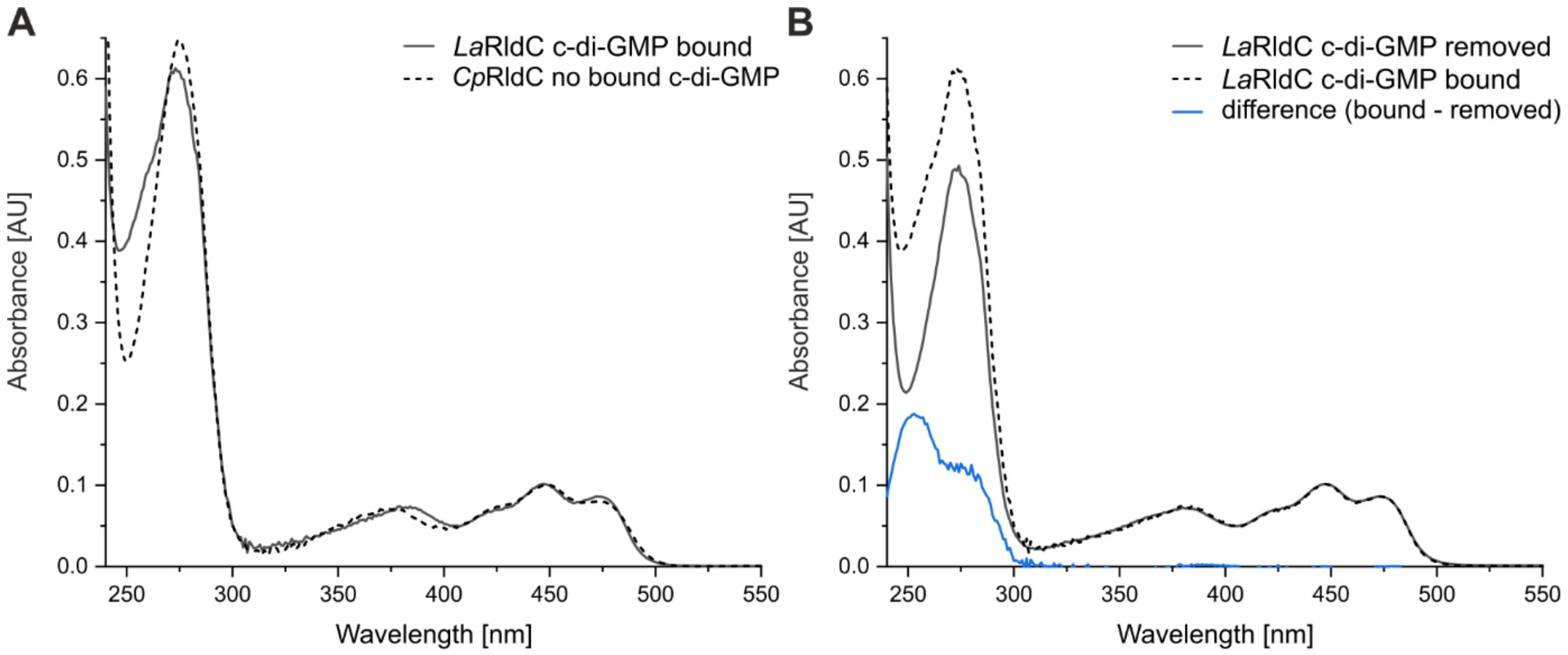

Figure S3: Absorption spectra identifying bound c-di-GMP. A) UV absorbance differs between *Cp*RldC (no bound c-di-GMP) and *La*RldC (bound c-di-GMP). B) Comparison of *La*RldC before and after removing bound c-di-GMP. The difference spectrum matches the spectrum of guanosine.

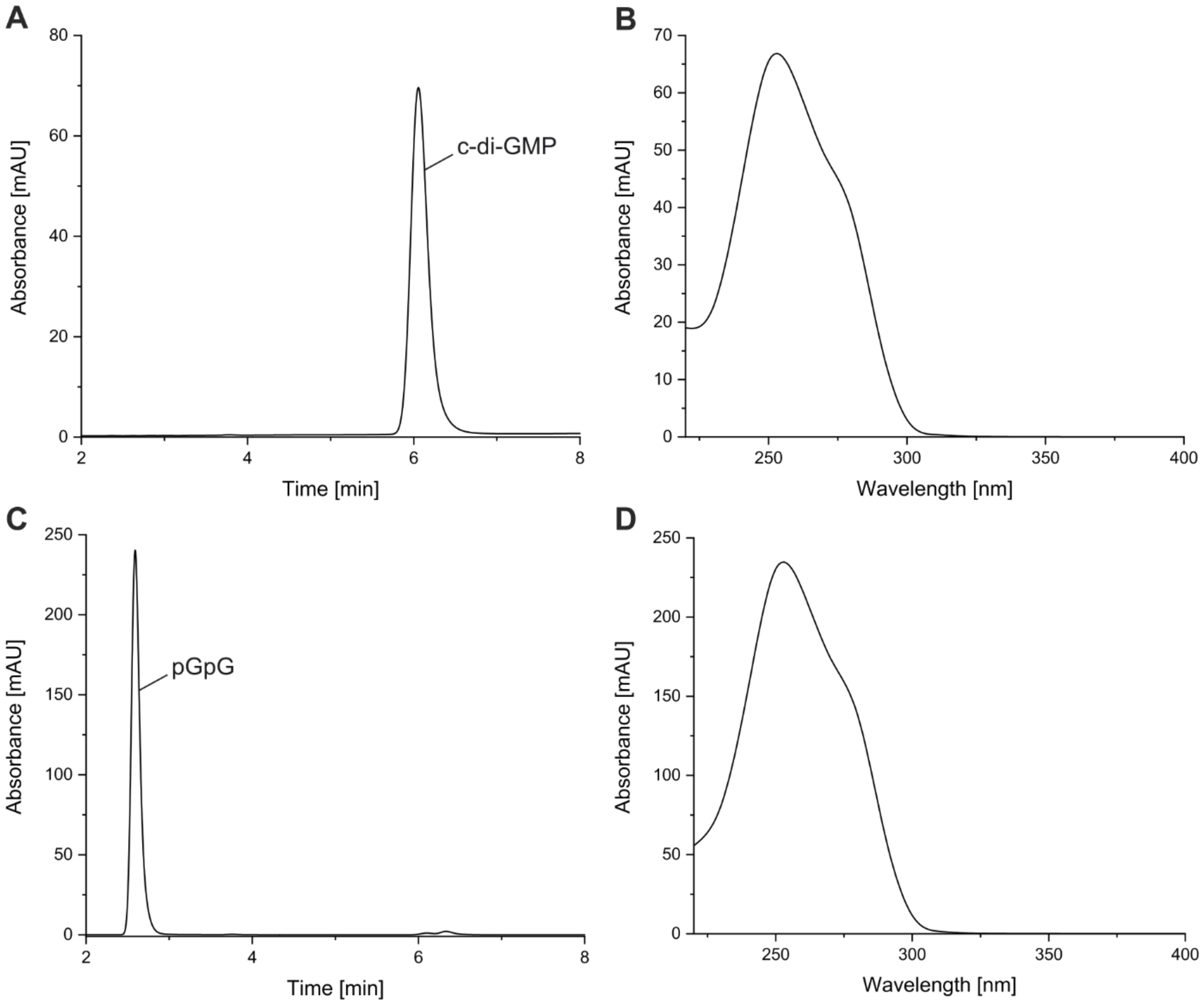

Figure S4: HPLC traces and peak spectra confirming bound or converted c-di-GMP. A) Denatured sample of *La*RldC showing bound c-di-GMP. B) Absorption spectrum of the peak visible in panel A. C) Denatured sample of *La*RldC after incubation with RocR showing conversion of c-di-GMP to pGpG. D) Absorption spectrum of the pGpG peak shown in C.

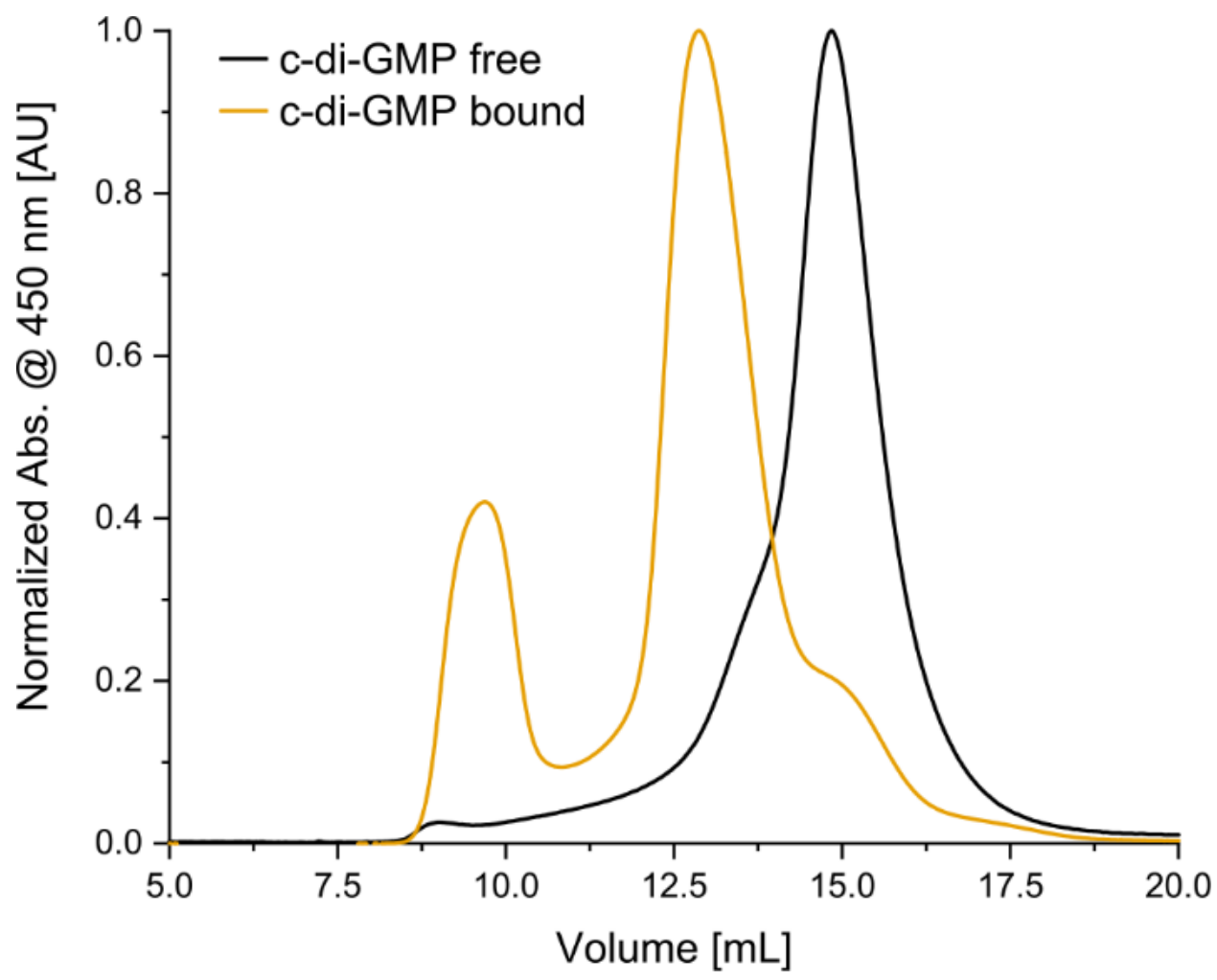

Figure S5: FPLC traces of the *Ro*RldC purification. The elution volume of the main peak changes once bound c-di-GMP is removed. Purification on a Cytiva S200 increase 10/300 column equilibrated in 10 mM Tris pH 8.0, 500 mM NaCl, 2 mM NaCl.

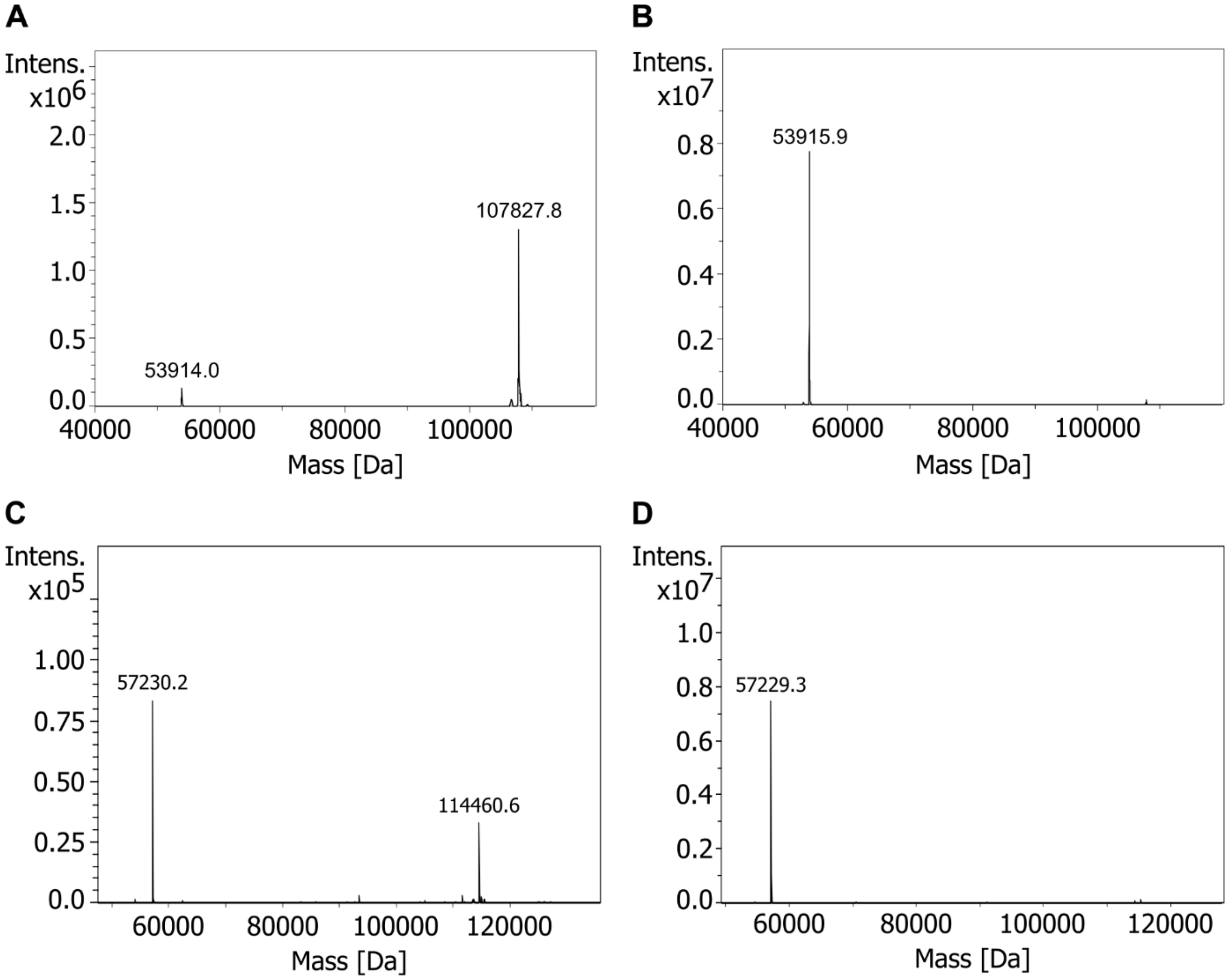

Figure S6: Intact Mass measurements of *La*RldC and *Ls*RldC. A) *La*RldC measured after removal of bound c-di-GMP. In addition to the monomer mass, a dimer is also present. The expected mass for the protein is 53,915.8 Da. B) Measurement of *La*RldC after the addition of TCEP to the buffer and detection of only the monomer's mass. The minor amount of dimer can be considered an artefact of maximum entropy deconvolution. C) *Ls*RldC after protein purification. Monomer and dimer peaks are visible. Expected monomer mass of 57,230.4 Da. D) Intact mass measurement of *Ls*RldC after addition of TCEP to the buffer. Only the peak corresponding to the monomer remains.

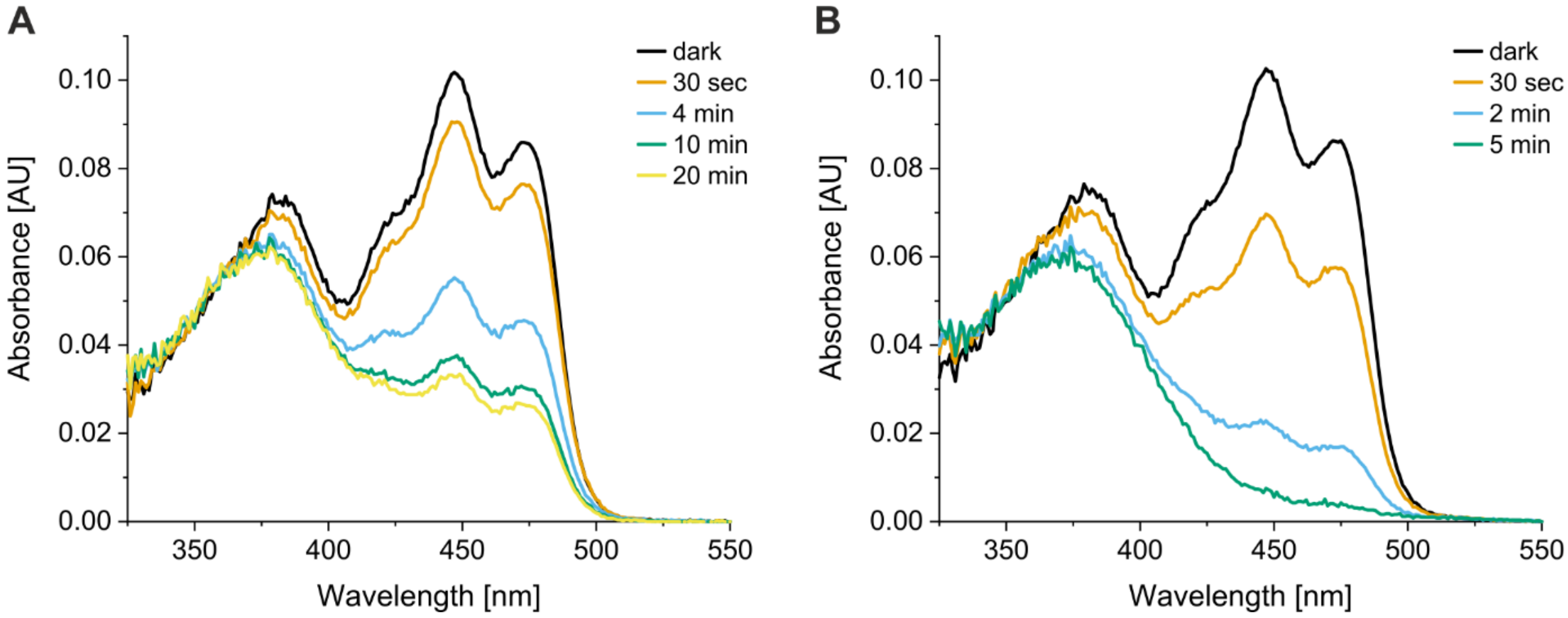

Figure S7: Influence of light quality on dark- to light-state transition of *La*RldC. A) Sample illuminated with 455 nm blue light LED (ThorLabs, 3 mW/cm^2) for indicated amounts of time. B) Illumination with 470 nm blue light LED (ThorLabs, 3 mW/cm^2) for specified times.

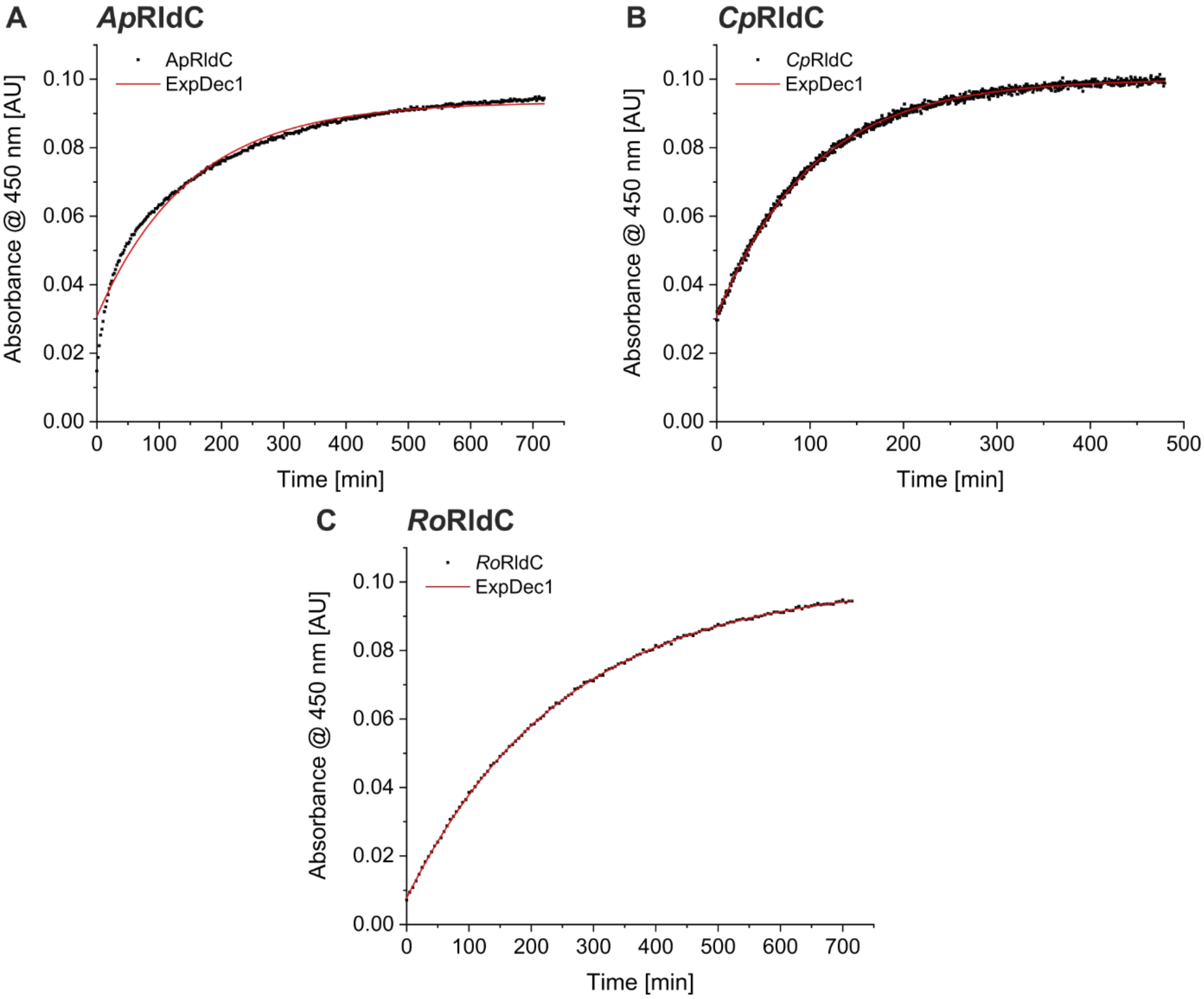

Figure S8: Single exponential fits of thermal recoveries. A) Shows *Ap*RldC recovery data (black) and exponential fit (red). Equation with rounded parameters y = -0.06 * $e^{(-x/150)}$ + 0.09; τ (mean lifetime) = 150 min. B) Recovery data of *Cp*RldC in black with calculated fit in red. y = -0.10 * $e^{(-x/101)}$ + 0.11; τ = 101 min. C) Measured recovery data of *Ro*RldC (black) with exponential fit (red). y = -0.09 * $e^{(-x/253)}$ + 0.1; τ = 253 min.

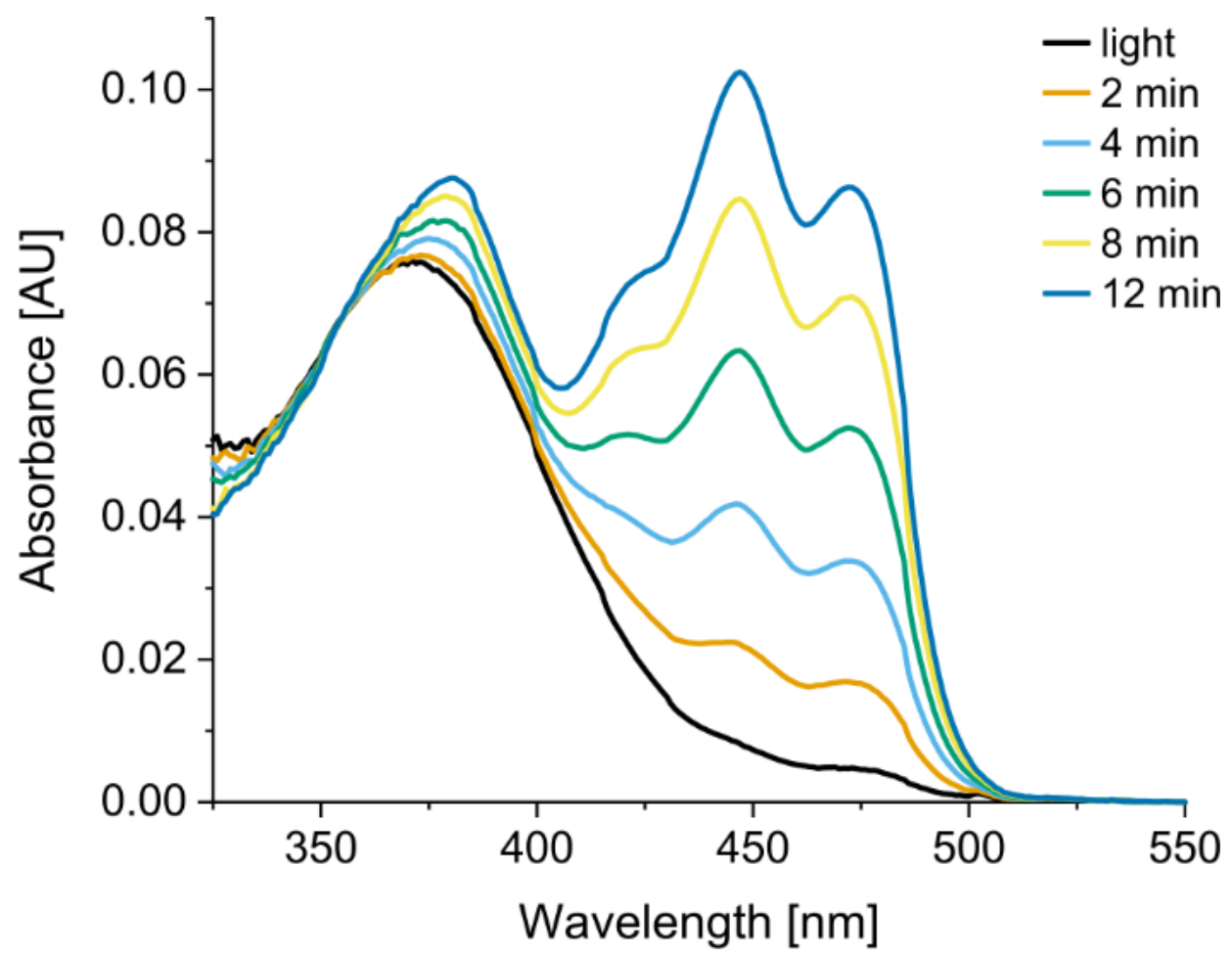

Figure S9: UV illumination of *La*RldC. Illumination with 395 nm light (2 mW/cm^2) switches the protein back to the dark state. Times of illumination are specified in the legend.

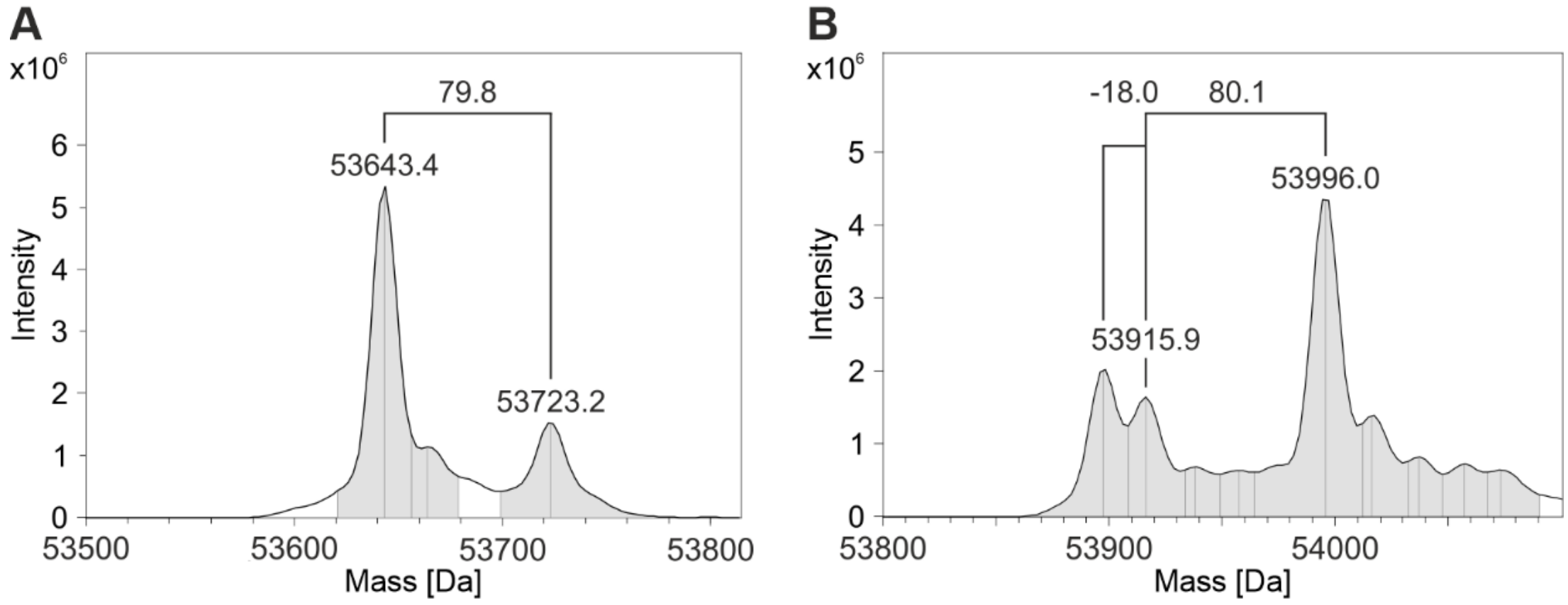

Figure S10: Intact mass measurement after incubation with 50 mM phosphoramidate. A) *Cp*RldC after multiple hours of incubation at 20 °C. Only moderate amounts of phosphorylated protein were detected. The approximately 80 Da mass difference is characteristic of protein phosphorylation. B) Mass spectrum showing phosphorylation of *La*RldC. The majority of the protein was phosphorylated after 1 h incubation at 20 °C. The peak at -18.0 Da is due to the neutral loss of H_3PO_4 from the phosphorylated species, a well-documented phenomenon in phosphorylated proteins [79, 80]

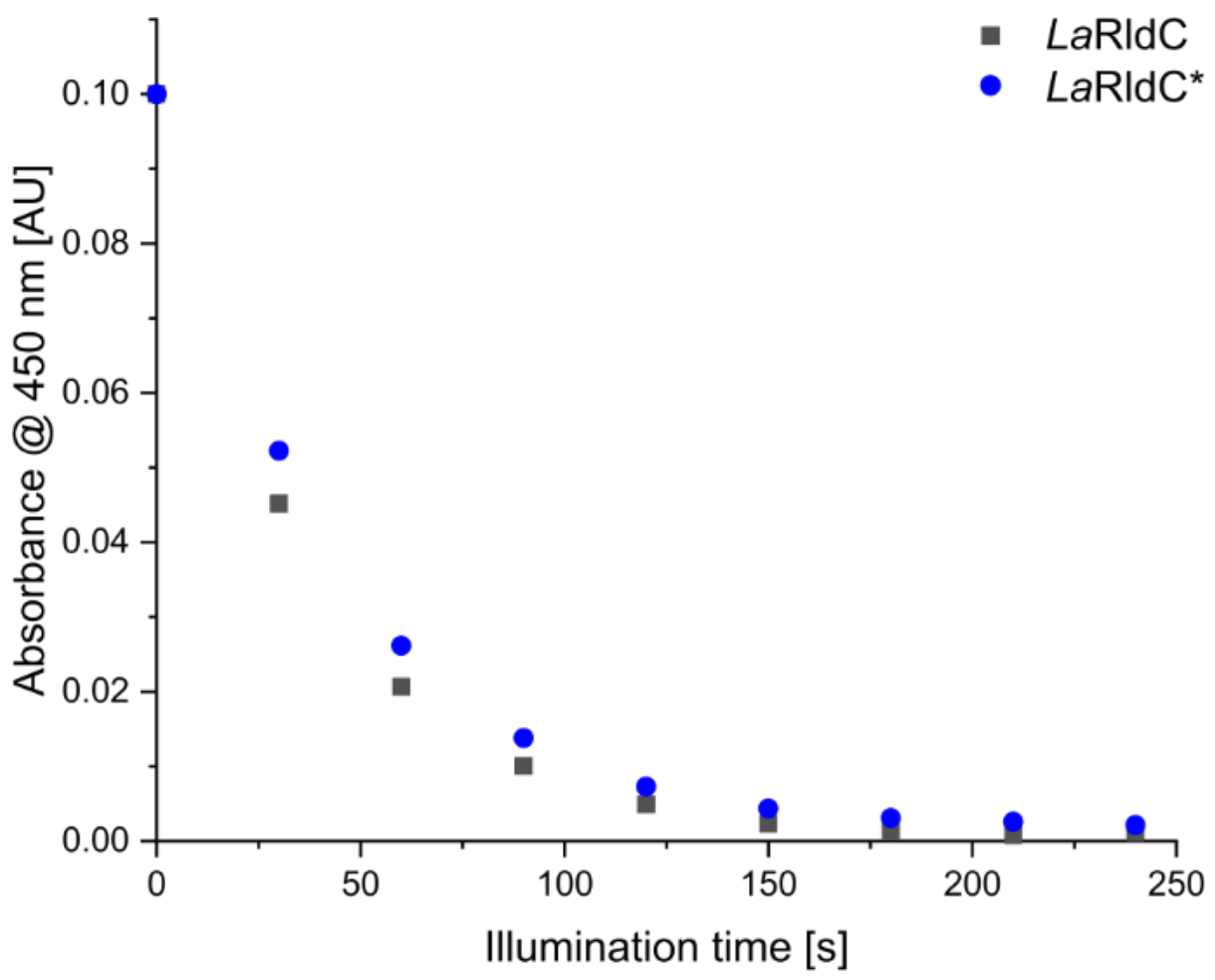

Figure S11: Activation of *La*RldC with and without phosphorylation. Shows the decrease of the 450 nm absorbance in response to illumination with 470 nm blue light. The phosphorylated sample indicated by * was incubated with 50 mM phosphoramidate before the measurement.

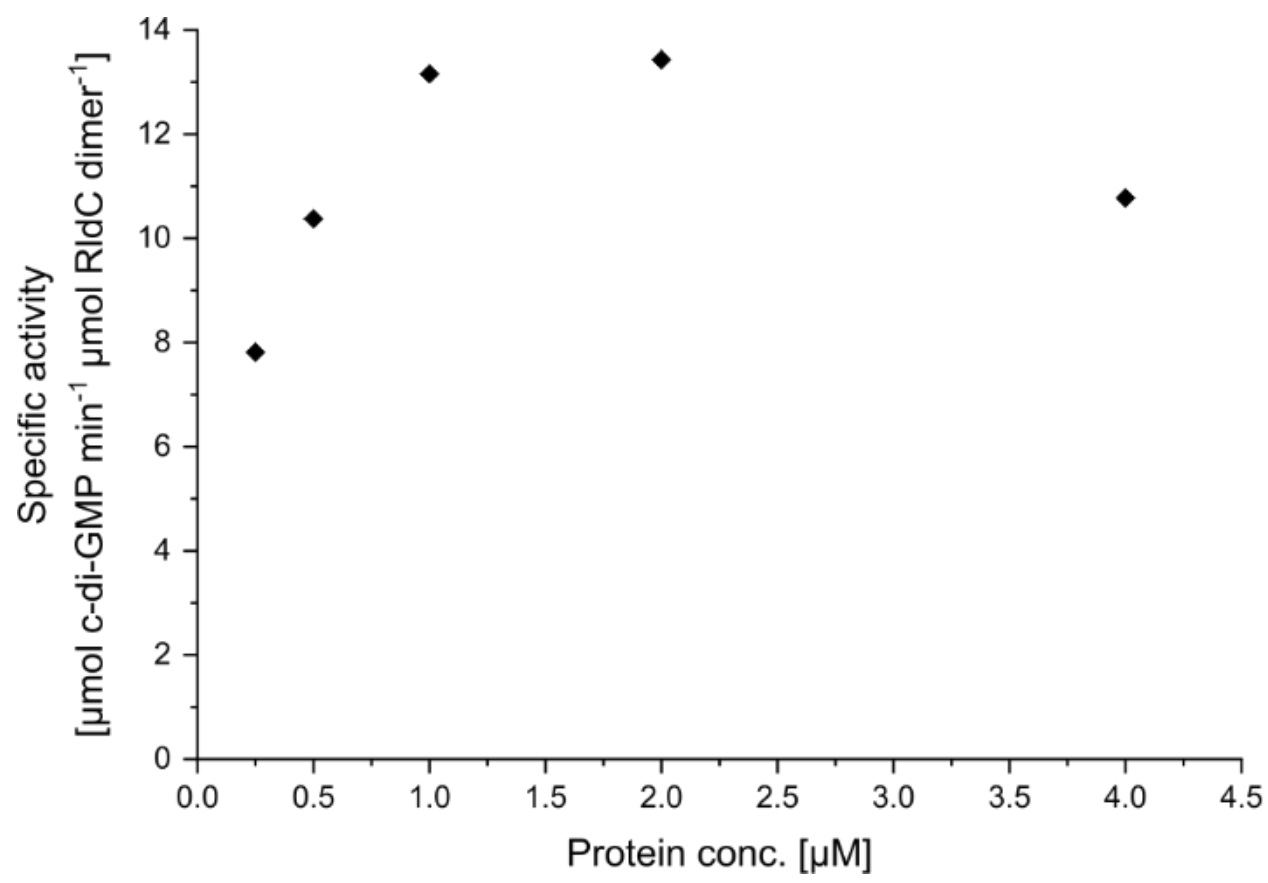

Figure S12: Concentration dependence of specific activity for *La*RldC. The data indicates that lower concentrations than 1 µM show lower specific activity. This suggests the possibility of a monomer-dimer equilibrium influencing the protein's activity. Concentrations above 2 µM are affected by high substrate turnover, thereby limiting the achievable specific activity.

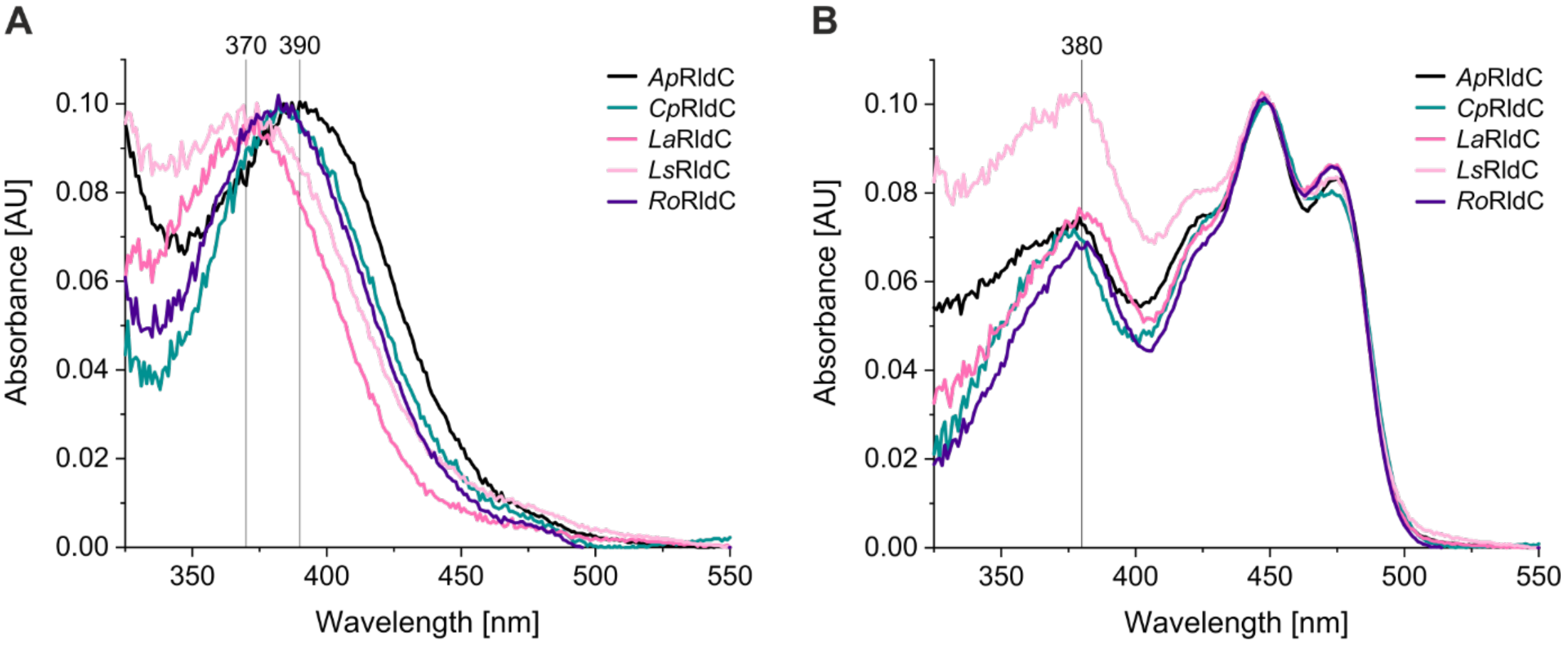

Figure S13: Differences in dark- and light-state absorption spectra of the studied RldC homologs. A) The light-state of *La*RldC and *Ls*RldC is significantly blue-shifted compared to the usual 390 nm maximum observed in other LOV domains. Spectra normalised based on the observed maximum. B) Normalised dark-state spectra of all soluble homologs, comparing the maximum of the UV-A band.

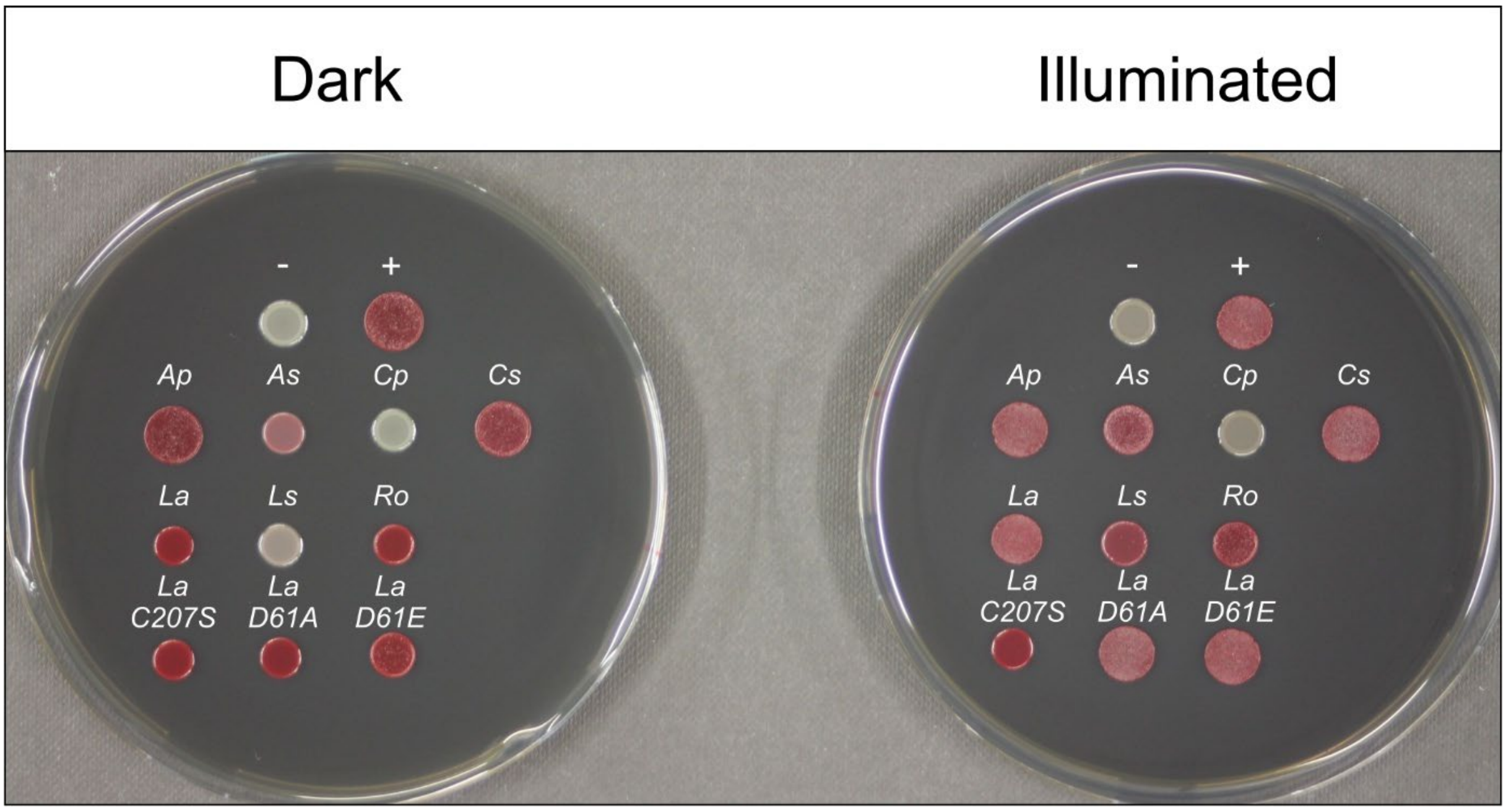

Figure S14: Uncut image of the in vivo screening plates shown in Figure 1.

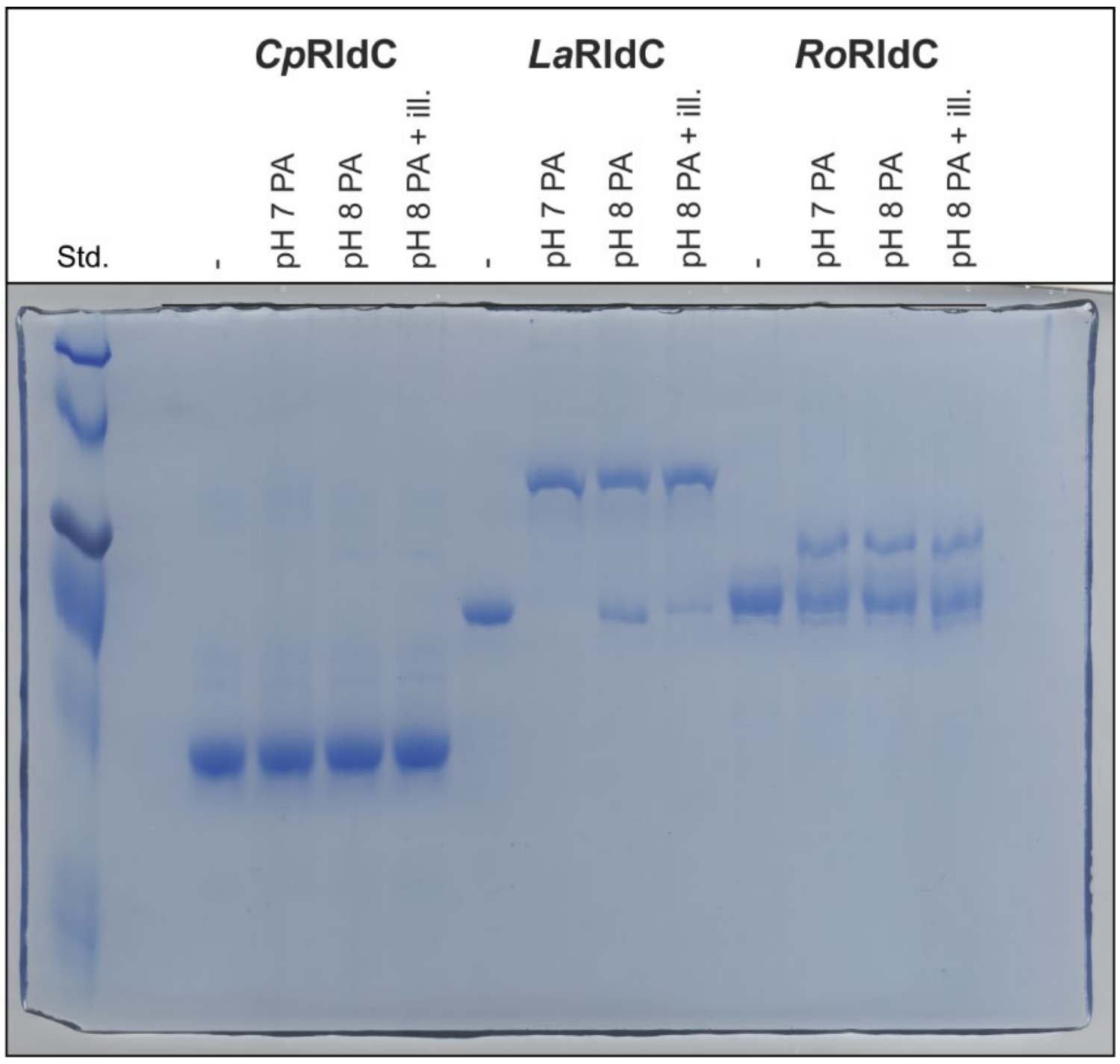

Figure S15: Complete Phos-Tag™ SDS-PAGE shown in Figure 5. The coloured protein standard was used to follow the progress of the gel once the running front left the gel. Due to the gel composition, the protein standard cannot be used to estimate molecular masses.

Table S1: Identifiers and background information on RldC homologs. Protein acronyms are built by adding the first letter of the genus and species to the RldC (Receiver LOV-activated diguanylate cyclase) abbreviation and were given to homologs selected for the study. Shades of grey highlight the proposed classification based on different linker lengths between the LOV and GGDEF domains.

Protein Acronym	Protein accession number	Organism	Genome entry	Isolation environment
*La*RldC	ERT09697.1	*Lyngbya aestuarii BL J*	AUZM01000002.1	marine
*Ls*RldC	WP_009786481.1	*Lyngbya sp. PCC 8106*	GCA_000169095.1	marine
	MGC9527602.1	*Limnospira sp.*	SAMN47943637	hot spring
	WP_324705780.1	*Lyngbya sp. CCY1209*	JAYFER010000546.1	marine
	WP_315686483.1	*Limnospira sp. PMC 1234.20*	GCA_032350155.1	brackish
*Ap*RldC	BAI88979.1	*Arthrospira platensis NIES-39*	AP026945.2	freshwater
*Cp*RldC	MBR8831174.1	*Chlorogloea purpurea*	JADQBB010000013.1	freshwater
	WP_073598543.1	*Hydrococcus rivularis NIES-593*	GCA_001904635.1	hot spring
	WP_217523204.1	*Pleurocapsa sp. PCC 7327*	GCA_000317025.1	hot spring
	WP_332863751.1	*Pannus brasiliensis*	GCA_036688125.1	freshwater
	WP_323301584.1	*Crocosphaera sp. UHCC 0190*	GCA_034932065.1	marine
	WP_323292403.1	*Crocosphaera sp. XPORK-15E*	GCA_034931425.1	marine
*As*RldC	WP_124976064.1	*Aphanothece sacrum* FPU1	BDQK01000016.1	freshwater
*Cs*RldC	HAC62459.1	*Cyanothece sp. UBA12306*	DLXW01000041.1	marine
*Ro*RldC	WP_012595352.1	*Rippkaea orientalis PCC 8801*	GCA_000021805.1	freshwater

Table S2: Data obtained from mass spectrometry measurements. Expected masses were calculated using Prot pi (www.protpi.ch/Calculator/ProteinTool). Holo dimer masses contain the average masses of 2 molecules of FMN in addition to two protein protomers. The measurement accuracy was calculated in ppm. Shades of grey highlight the classification by LOV-GGDEF linker length.

	Native MS			Intact Mass		
**Protein**	**Expected Mass (holo dimer) [Da]**	**Measured Mass [Da]**	**Accuracy [ppm]**	**Expected Mass [Da]**	**Measured Mass [Da]**	**Accuracy [ppm]**
***La*RldC**	108,744.3	108,747.0	24.8	53,915.8	53,915.7	-1.9
***Ls*RldC**	115,373.5	115,376.5	26.0	57,230.4	57,230.3	-1.7
***Ap*RldC**	114,164.1	114,165.3	10.5	56,625.7	56,623.2	-44.2
***Cp*RldC**	108,200.0	108,201.6	14.8	53,643.7	53,643.1	-11.2
***Ro*RldC**	110,761.1	110,763.2	19.0	54,924.2	54,923.6	-10.9

Table S3: Storage buffer composition for each of the studied homologs. Shades of grey highlight the classification by LOV-GGDEF linker length.

	Buffer composition			
***La*RldC**	10 mM Tris pH 8.0	500 mM NaCl	2 mM $MgCl_2$	1 mM TCEP
***Ls*RldC**	10 mM Tris pH 8.0	500 mM NaCl	2 mM $MgCl_2$	1 mM TCEP
***Ap*RldC**	10 mM Tris pH 8.0	50 mM NaCl	2 mM $MgCl_2$	
***Cp*RldC**	10 mM Hepes pH 7.0	500 mM NaCl	2 mM $MgCl_2$	
***Ro*RldC**	10 mM Tris pH 8.0	500 mM NaCl	2 mM $MgCl_2$	

Table S4: Percentage identity based on pairwise alignment of the studied homologs' amino acid sequences. Values are given in %. Shades of grey highlight the classification by LOV-GGDEF linker lengths. Cell colouring was generated using Excel's three-colour conditional formatting scale, with fixed thresholds of 15%, 55%, and 95%.

	***La*RldC**	***Ls*RldC**	***Ap*RldC**	***Cp*RldC**	***As*RldC**	***Cs*RldC**	***Ro*RldC**
***La*RldC**		87.97	50.2	44.54	52.71	51.98	52.07
***Ls*RldC**	87.97		49.8	44.54	53.85	51.77	53.42
***Ap*RldC**	50.2	49.8		46.04	50.21	49.48	50.94
***Cp*RldC**	44.54	44.54	46.04		48.54	51.36	48.86
***As*RldC**	52.71	53.85	50.21	48.54		67.64	75.21
***Cs*RldC**	51.98	51.77	49.48	51.36	67.64		75.57
***Ro*RldC**	52.07	53.42	50.94	48.86	75.21	75.57	

Table S5: Percentage identity based on pairwise alignment of individual domains and functional elements. Domain and linker boundaries are visible in Figure S1. Values are given in %. Shades of grey highlight the classification by LOV-GGDEF linker length. Cell colouring was generated using Excel's three-colour conditional formatting scale, with fixed thresholds of 15%, 55%, and 95%.

**Rec**							
	La	***Ls***	***Ap***	***Cp***	***As***	***Cs***	***Ro***
La		90.77	50	35.16	53.13	51.54	52.71
Ls	90.77		49.23	35.94	53.49	53.85	56.92
Ap	50	49.23		36.15	52.34	50	51.56
Cp	35.16	35.94	36.15		38.28	40.63	38.28
As	53.13	53.49	52.34	38.28		67.19	71.88
Cs	51.54	53.85	50	40.63	67.19		77.34
Ro	52.71	56.92	51.56	38.28	71.88	77.34	
**Rec-LOV linker**							
	La	***Ls***	***Ap***	***Cp***	***As***	***Cs***	***Ro***
La		72	48	16	24	28	16
Ls	72		40	16	16	24	24
Ap	48	40		36	21.88	31.25	31.25
Cp	16	16	36		23.33	33.33	23.33
As	24	16	21.88	23.33		50	62.5
Cs	28	24	31.25	33.33	50		68.75
Ro	16	24	31.25	23.33	62.5	68.75	
**LOV**							
	La	***Ls***	***Ap***	***Cp***	***As***	***Cs***	***Ro***
La		92.5	58.33	51.67	55.83	55.83	57.5
Ls	92.5		57.5	50	56.67	55.83	59.17
Ap	58.33	57.5		50.83	51.67	53.33	54.17
Cp	51.67	50	50.83		56.67	58.33	54.17
As	55.83	56.67	51.67	56.67		71.67	82.5
Cs	55.83	55.83	53.33	58.33	71.67		75
Ro	57.5	59.17	54.17	54.17	82.5	75	
**LOV-GGDEF linker**							
	La	***Ls***	***Ap***	***Cp***	***As***	***Cs***	***Ro***
La		76.19	38.1	23.81	38.1	52.38	42.86
Ls	76.19		42.86	23.81	47.62	42.86	42.86
Ap	38.1	42.86		57.14	35.71	39.29	39.29
Cp	23.81	23.81	57.14		46.43	50	53.57
As	38.1	47.62	35.71	46.43		74.29	88.57
Cs	52.38	42.86	39.29	50	74.29		80
Ro	42.86	42.86	39.29	53.57	88.57	80	
**GGDEF**							
	La	***Ls***	***Ap***	***Cp***	***As***	***Cs***	***Ro***
La		86.52	50.56	52.35	61.82	59.15	58.43
Ls	86.52		50.53	52.94	63.64	57.93	58.43
Ap	50.56	50.53		52.35	56.36	52.44	54.82
Cp	52.35	52.94	52.35		56.36	59.15	57.83
As	61.82	63.64	56.36	56.36		67.07	72.12
Cs	59.15	57.93	52.44	59.15	67.07		75
Ro	58.43	58.43	54.82	57.83	72.12	75	

Supplementary references

[79] Wolf D. Lehmann, Ralf Krüger, Mogjiborahman Salek, Chien-Wen Hung, Florian Wolschin, Wolfram Weckwerth, "Neutral loss-based phosphopeptide recognition: a collection of caveats", Journal of proteome research, Vol. 6, No. 7, pp. 2866–2873, 2007.

[80] Li Cui, Ipek Yapici, Babak Borhan, Gavin E. Reid, "Quantification of competing H3PO4 versus HPO3 + H2O neutral losses from regioselective 18O-labeled phosphopeptides", Journal of the American Society for Mass Spectrometry, Vol. 25, No. 1, pp. 141–148, 2014.
